# An investigation of the effects of meteorological factors on the incidence of tuberculosis

**DOI:** 10.1038/s41598-024-52278-y

**Published:** 2024-01-24

**Authors:** Minli Chang, Mawlanjan Emam, Xiaodie Chen, Dongmei Lu, Liping Zhang, Yanling Zheng

**Affiliations:** 1 College of Public Health, Xinjiang Medical University, Ürümqi, 830017 People’s Republic of China; 2 Center for Disease Control and Prevention, Kashgar, People’s Republic of China; 3 Center of Pulmonary and Critical Care Medicine, People’s Hospital of Xinjiang Uygur Autonomous Region, Ürümqi, People’s Republic of China; 4 College of Medical Engineering and Technology, Xinjiang Medical University, Ürümqi, 830017 People’s Republic of China

**Keywords:** Diseases, Risk factors

## Abstract

To explore the influence of meteorological factors on the incidence of tuberculosis (TB) in Yingjisha County, Kashgar Region, Xinjiang, and to provide a scientific basis for the prevention and control of TB. The Spearman correlation analysis and distribution lag nonlinear model analysis were conducted on the number of daily reported cases of TB from 2016 to 2023 to study the association effect of various meteorological factors and the daily incidence number of TB in Yingjisha County. A total of 13,288 TB cases were reported from January 2016 to June 2023, and June to October is the peak period of annual TB incidence. Spearman correlation analysis revealed that average daily temperature (AT) and average daily wind speed (WS) were positively correlated with TB incidence (r_AT_ = 0.110, r_WS_ = 0.090); and average daily relative humidity (RH) and TB incidence was negatively correlated (r_RH_ = − 0.093). When AT was − 15 °C, the RR reached a maximum of 2.20 (95% CI: 0.77–6.29) at a lag of 21 days. When RH was 92%, the RR reached a maximum of 1.05 (95% CI: 0.92–1.19) at a lag of 6 days. When WS was 5.2 m/s, the RR reached a maximum of 1.30 (95% CI: 0.78–2.16) at a lag of 16 days. There is a non-linearity and a certain lag between meteorological factors and the occurrence and prevalence of TB in the population, which is mainly manifested in the fact that the risk of incidence of TB decreases with the increase of the daily AT, has a hazardous effect within a certain range of humidity as the average daily RH rises, and gradually increases with the increase of the average daily WS. Local residents are advised to pay attention to climate change so as to take appropriate preventive measures, especially women and middle and old age group should pay close attention to climate change and add more clothes in time, minimise travelling in hazy weather and windy and sandy weather, maintain good nutrition, adequate sleep and moderate exercise in daily life to enhance their immunity, wash hands frequently and ventilate the air, and try to avoid staying in humid and confined spaces in order to reduce the risk of latent TB patients developing the disease.

## Introduction

Pulmonary TB, Broadly speaking, it is a common serious respiratory infectious disease triggered by incidence of the lungs with Mycobacterium Pulmonary TB^1^. Mycobacterium TB spreads widely in the air through the medium of large quantities of droplets brought out by sneezing, coughing, sputum coughing, or loud talking of TB patients, and the human body becomes infected and suffered from TB when inhaled^2^. Due to its uncontrollable ways of transmission, the disease has highly contagious, and once inadvertently infected, there is a certain rate of recurrence after treatment, which is the main reason why TB has been with us for thousands of years^3^. In recent years, a large number of studies at domestic and abroad have shown that there is a correlation between the incidence of TB and meteorological factors, which may influence the survival and spread of the Mycobacterium to a certain extent. Due to China's vast territory, its boundaries spanning from east to west, with a total area of 10.45 million square kilometers, it possesses a wide variety of topographical features and climate types. From mountains to plains, from lakes to rivers, from subtropical to frigid zones, it has almost all kinds of natural landscapes and climate types in the world. The influence of meteorological factors on the incidence effect of TB therefore varies^4,5^. Yingjisha County, Xinjiang, is one of the regions with high incidence of TB in China, and the study of the influence of meteorological factors on the incidence of TB in this region has a relatively good reference value for TB prevention and control.

Unavoidable climate change is caused by high levels of anthropogenic greenhouse gas emissions, to some extent, directly or indirectly increases exposure to risk factors for diseases of the respiratory system, thus posing a significant threat to respiratory health, directly or indirectly^6^. Climate change is therefore a global public health challenge. It can impact the burden of TB in multiple ways^7^. Khaliq A et al. found a significant correlation between temperature and TB incidence by studying the relationship between TB and temperature in Lahore, Pakistan, 2006–2013^8^. Zhang et al. conducted a systematic analysis of the incidence of TB in Beijing based on structural equation modelling, and they found that temperature and wind speed positively affected the incidence of TB by increasing the concentrations of inhalable fine particles and SO_2_, while precipitation, atmospheric pressure and relative humidity negatively affected the incidence of TB by indirectly decreasing the concentrations of inhalable fine particles and SO_2_^9^. Rao HX et al. applied a spatial panel data model and found that the incidence of TB was positively correlated with temperature, precipitation and wind speed, and that the incidence of TB decreased by 9% and 3%, and increased by 7%, for each increase in temperature, precipitation and wind speed of 10 °C, 2 cm and 1 m/s, respectively^10^. Deng B et al. applied a BP artificial neural network model to explore the influence of meteorological factors on the incidence of TB and found that the average evaporation had the greatest influence on the incidence of TB, and the average atmospheric pressure also had a certain influence^11^. Cao Fu et al. used a single-pollutant-temperature interaction model to analyze the role of air pollutants and temperature on the risk of TB incidence, and found that high-temperature exposure or low-temperature exposure could cause an increased risk of TB incidence in Yulin City^12^. DING Fan et al. explored the effects of different meteorological factors on the incidence of TB using conditional Poisson regression with a spatial–temporal stratified case-crossover design found that the temperature and the incidence of TB were correlated in Ningxia, and that a decrease in the temperature might increase the risk of the incidence of TB, with a greater effect on the incidence of females and the age group of 36–64 years old^13^. Xiao et al. analyzed 10 consecutive years of TB surveillance data in southwestern China using DLNM and found that average temperature was negatively associated with TB incidence with a lag of 2 months. Total precipitation and minimum relative humidity were also negatively correlated with TB incidence with a lag of 3 and 4 months, respectively, and average wind speed and total sunshine hours showed an immediate rather than a lagged effect on TB incidence^14^. Huang et al. used DLNM to study the relationship between daily TB hospitalizations and meteorological factors and air pollutants in 16 cities in Anhui Province and found that low temperature exposure increased the risk of TB hospitalizations at lags of 0 and 1 day, and that associations between temperature and TB admissions varied according to air pollution, latitude and healthcare resources. The overall exposure–response curve between relative humidity and TB was almost a flat line with 78% of the reference relative humidity^15^. Wang et al. found short-term lag and cumulative lag effects in the development of TB using the DLNM^16^. From the above studies, it can be concluded that the lag effect of climate change on TB exists, and DLNM can better analyze the impact of meteorological factors on the incidence of TB, and explore the non-linear exposure–response dependence and delayed effect of the relationship between meteorological factors and TB incidence^17^. At this stage, the task of TB prevention and control in China is still arduous, and Yingjisha, as one of the areas with high incidence of patients with TB in China, due to its complex geographical environment, the meteorological conditions of Yingjisha are also very unique. Therefore, the prevention strategies given in previous studies are not applicable to the residents of Yingjisha County. And in the existing studies lacks a detailed discussion on the relationship between its climate change and the incidence in patients with TB in various populations. We therefore investigated the effect of meteorological factors on the local transmission of TB in Yingjisha County.

The purpose of this study was to use lag nonlinear model of distribution (distributed lag non-linear model, DLNM) to analyze the association of meteorological factors and TB in Yingjisha County from January 2016 to June 2023, and to explore the influence of meteorological factors on the incidence of TB in patients with different gender and age groups. It is hoped that the results can provide reference for local residents to take corresponding preventive measures through climate change to reduce the risk of incidence of TB.

## Material and methods

### Study area

Yingjisha County is located in the southwestern part of Xinjiang Uygur Autonomous Region, at the northern foot of Kunlun Mountain and the western edge of Tarim Basin, with a total area of 3425 square kilometers, 4 towns and 10 townships under the jurisdiction of 178 administrative villages (including 15 subdivided villages), 19 communities, and 4 state-run agricultural, forestry and animal husbandry farms, with a cultivated area of 450,000 acres. The resident population is 276,641.

### Data collection

The number of reported cases of TB in Yingjisha County, Kashgar Region, Xinjiang Uygur Autonomous Region, from 1 January 2016 to 30 June 2023 was obtained from the China Infectious Disease Network Reporting System. Meteorological data of the region for the same period were obtained from the China Meteorological Data Network, including average daily temperature (AT, °C), average daily relative humidity (RH, %), and average daily wind speed (WS, m/s).

### Statistical method

In this study, Excel 2021 software was applied for data preprocessing, R4.1.3 software was used for descriptive statistical analysis of the data and dlnm2.4.2 software package was used to fit distributed lagged nonlinear model to analyze the effective relationship between each meteorological factor and the incidence of TB, and Spearman correlation analysis was carried out between each meteorological factor and the number of daily incidence of TB using SPSS 25 software. The test level was 0.05.

The core idea of DLNM is to add lag dimension to the exposure–response relationship between each meteorological factor and the health effects of each population by ways of cross-basis functions^18^, establishing cross-basis functions for the variables and introduce a generalize linear model, so as to describe the distribution of the changes of their effects in the independent variable dimension and lag dimension at the same time. The effects of meteorological factors on human health present a nonlinear (e.g., J, V, or U-shape relationship) and there are lagged effects^17^. Since the number of daily incidence cases of TB does not obey normal distribution, which is mostly zero and discrete, “quasi-Poisson” was used as the connecting function. In this study, the cross-basis of average daily temperature, average daily relative humidity, and average daily wind speed were used as independent variables, and the number of daily incidence cases of TB in different populations was used as the dependent variable, while controlling for the influences of average daily relative humidity and average daily wind speed, average daily temperature and average daily wind speed, average daily temperature and average daily relative humidity. The time trend, and the day of the week effects, which were included as confounders in the model, and the basic model was as follows:

$$\begin{gathered} \log \left[ {{\text{E}}\left( {Y_{t} } \right)} \right] = \alpha + \beta AT_{t,l} + {\text{ns}}\left( {RH_{t} ,df = 3} \right) + {\text{ns}}\left( {WS_{t} ,df = 3} \right) + {\text{ns}}\left( {Time,df = 7/year} \right) + \gamma Dow_{t} \hfill \\ \log \left[ {{\text{E}}\left( {Y_{t} } \right)} \right] = \alpha + \beta RH_{t,l} + {\text{ns}}\left( {AT_{t} ,df = 3} \right) + {\text{ns}}\left( {WS_{t} ,df = 3} \right) + {\text{ns}}\left( {Time,df = 7/year} \right) + \gamma Dow_{t} \hfill \\ \log \left[ {{\text{E}}\left( {Y_{t} } \right)} \right] = \alpha + \beta WS_{t,l} + {\text{ns}}\left( {AT_{t} ,df = 3} \right) + {\text{ns}}\left( {RH_{t} ,df = 3} \right) + {\text{ns}}\left( {Time,df = 7/year} \right) + \gamma Dow_{t} \hfill \\ \end{gathered}$$

where ${Y}_{t}$ is the number of pulmonary TB cases on day $t$; $\alpha$ is the intercept, $AT_{t,l}$, $RH_{t,l}$, $WS_{t,l}$ is the two-dimensional matrix representing the average index of each meteorological factor based on the “cross-base” function in DLNM, $\beta$ is the explanatory variable coefficient of the two-dimensional matrix of the average index of each meteorological factor, and $l$ is the number of days delayed. $ns$ is a natural cubic spline function, $ns({AT}_{t},df=3)$ refers to the use of a natural cubic spline function with 3 degrees of freedom to control the influence of the average daily temperature, where $df$ is the degree of freedom; $ns({RH}_{t},df=3)$ refers to the use of a natural cubic spline function with 3 degrees of freedom to control the influence of the daily average humidity; $ns({WS}_{t},df=3)$ refers to the use of a natural cubic spline function with 3 degrees of freedom to control the influence of the average daily wind speed; $ns(Time,df=3)$ refers to the use of natural cubic spline functions with annual degrees of freedom equal to 7 to control the effects of long-term trends and seasonality, ${Dow}_{t}$ refers to the dummy variable whose day $t$ is the day of the week.

With reference to previous studies, the corresponding degrees of freedom were selected as model parameters according to the characteristics of TB and the principle of minimum AIC value^19,20^, and the degrees of freedom of the natural spline function for average daily temperature, average daily relative humidity, average daily wind speed and lag were finally set to be 4 and 3, respectively, through the model testing; the natural degree of the long-term trend was set to be 7/year, and the maximum lag was set to be three weeks, and the model was constructed with 15.3 °C (annual average temperature), 43% (annual average relative humidity), and 0.8 m/s (annual average wind speed) as reference, respectively. P_10_ = − 3.6 °C, P_90_ = 26.7 °C; P_10_ = 26%, P_90_ = 68%; P_10_ = 0.4 m/s, P_90_ = 1.6 m/s were used as the nodes of low and high level meteorological factors to analyze the association effects of different temperatures, relative humidity, and wind speeds on the incidence of TB at a lag time of 0–21 days, and the cumulative relative hazard RR (95% CI) values were calculated, and also analyzed the effect of each meteorological factor on the number of daily TB incidence after gender and age stratification.

### Ethics approval and consent to participate

This research did not require institutional review approval since all data were publicly available and collected from existing online databases. This research did not involve any human subjects.

## Results

### Incidence characteristics of TB

**Figure 1:**
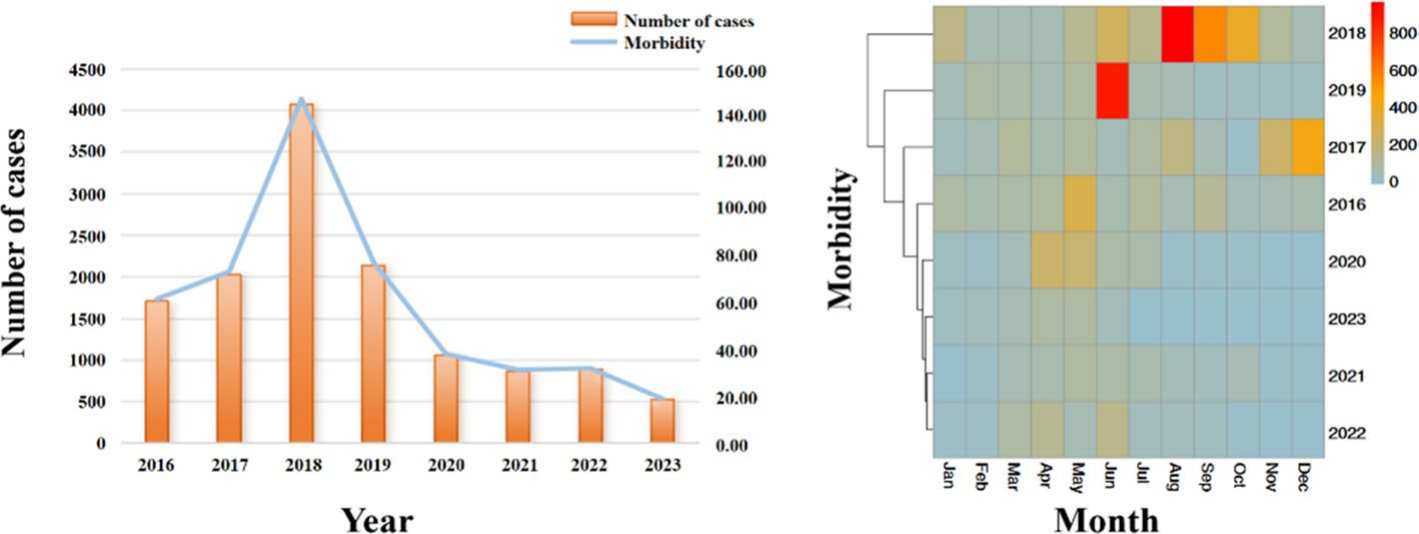
Time distribution trend of pulmonary tuberculosis in Yingjisha County from 2016 to 2023.

A total of 13,288 cases of TB were reported in Yingjisha County from 1 January 2016 to 30 June 2023. The incidence of TB from January 2016 to June 2023 showed a “jagged” development trend, with 2 peaks of incidence, namely the 1st peak of incidence in 2018, with an incidence rate of 14.72/10,000, followed by the 2nd peak of incidence in 2022, with an incidence rate of 3.21/10,000. This is partly due to fluctuations in meteorological factor. The emergence of the first peak is mainly due to the efforts to further reduce the harm caused by TB and accelerate the construction of a healthy China, with the goal of ending TB as a public health challenge by 2030. Kashgar, Xinjiang has actively responded to the national call and resolutely implemented the 13th Five-Year Plan for TB control. On the basis of the “12th Five-Year Plan” work, we will adhere to the principle of putting prevention first and combining prevention and treatment. Increasing the active screening of TB patients, so the number of reported cases in 2017 increased compared to 2016 and peaked in 2018. The number of reported cases in the second small peak was not much different from that in 2021 and 2023, which was largely due to the fluctuation of meteorological factors and novel coronavirus. The incidence of TB in Yingjisha County occurs throughout the year, and the incidence of TB shows seasonal distribution, with the peak incidence occurring in the months of June to October, as shown in Fig. 1.This may be due to the special geographical location of Yingjisha County causing its longer summer months. In the case of rising temperature and increasing humidity in summer, Mycobacterium TB is active, which is conducive to the spread of TB. In addition, the frequent population activity in summer and autumn and increasing human contact may also accelerate the spread of TB. The effects of climatic conditions and population activity may have contributed to the high incidence of TB during this time period.

### Descriptive statistical analysis of meteorological factors and daily incidence of TB

**Figure 2:**
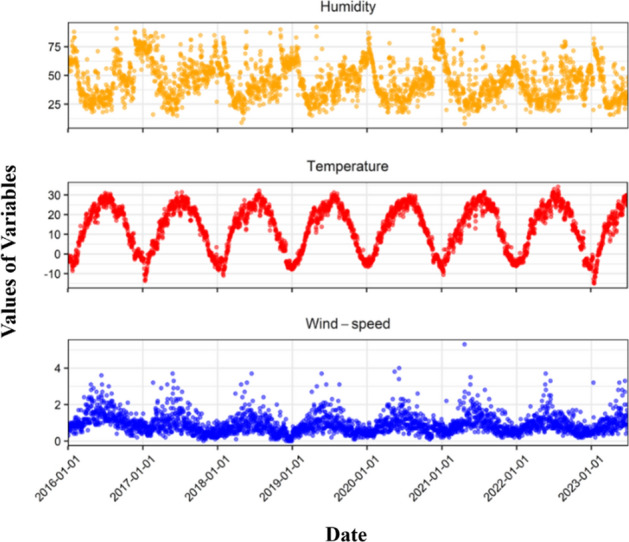
Time trends of meteorological factors in Yingjisha County, 2016–2023.

Of the 13,288 cases of TB reported in Yingjisha County in 2016–2023, 6342 (48%) were males and 6946 (52%) were females; 1182 (10%) were the age group of ≤ 35 years old, 5702 (43%) were the age group of 36–64 years old, and 6282 (47%) were the age group of ≥ 65 years old, and the highest daily incidence of TB was 344 cases, with an average daily incidence was 4.8 cases/day. The median average daily temperature, average daily relative humidity and average daily wind speed were 15.3 °C, 43% and 0.8 m/s, respectively, as shown in Table 1, and the temporal distribution graph showed a certain periodicity, as shown in Fig. 2.

**Table 1:** Description of daily incidence and meteorological factors of pulmonary TB in Yingjisha County.

	Daily cases						Meteorological factor		
	Total	Male	Female	≤ 35	36–64	≥ 65	AT (°C)	RH (%)	WS (m/s)
Min	0	0	0	0	0	0	− 15	8	0
P_1_	0	0	0	0	0	0	− 8.9	18	0.1
P_5_	0	0	0	0	0	0	− 5.6	23	0.3
P_10_	0	0	0	0	0	0	− 3.6	26	0.4
P_25_	0	0	0	0	0	0	2.6	32.5	0.6
P_50_	1	0	0	0	0	0	15.3	43	0.8
P_75_	4	2	2	0	2	2	23.0	56	1.2
P_90_	11	5	6	1	5	5	26.7	68	1.6
P_95_	19	9.15	11	2	8	9	28.1	74	2
P_99_	61.6	32	32.6	6	27.6	33.6	30.3	84	2.9
Max	344	165	179	26	189	152	34.1	92	5.3
$\overline{x }$±s	4.80 ± 13.71	2.30 ± 6.78	2.54 ± 7.27	0.40 ± 1.31	2.00 ± 6.31	2.29 ± 7.22	13.10 ± 11.41	45.2 ± 15.85	0.93 ± 0.55

### Analysis of the correlation between meteorological factors and the daily incidence of TB

Spearman correlation analysis of the daily incidence data of TB for each meteorological factor and for the same period from January 2016 to June 2023 in Yingjisha County showed that the correlation between the number of daily reported incidence of TB and each meteorological factor was statistically significant (*P* < 0.05). Both average daily temperature and average daily wind speed were positively correlated with daily TB incidence data (r_AT_ = 0.110, r_WS_ = 0.090), and average daily relative humidity was negatively correlated with daily TB incidence data (r_RH_ = − 0.093). The most significant effect of average daily temperature on the incidence of TB in the whole population was found (r_AT_ = 0.110), as shown in Table 2.

**Table 2:** Demographic and daily meteorological data characteristics in Yingjisha County from 2016 to 2023.

	AT	RH	WS	Total	Male	Female	≤ 35	36–64
RH	− 0.595**							
WS	0.523**	− 0.422**						
Total	0.110**	− 0.093**	0.090**					
Male	0.098**	− 0.078**	0.095**	0.907**				
Female	0.117**	− 0.088**	0.081**	0.927**	0.733**			
≤ 35	0.097**	− 0.039*	0.088**	0.590**	0.564**	0.553**		
36–64	0.109**	− 0.088**	0.089**	0.897**	0.829**	0.851**	0.470**	
≥ 65	0.095**	− 0.083**	0.078**	0.894**	0.829**	0.838**	0.445**	0.715**

*p < 0.05, **p < 0.01.

### DLNM analysis of the impact of various meteorological factors on TB incidence

#### Effect of AT on the incidence of TB

**Figure 3:**
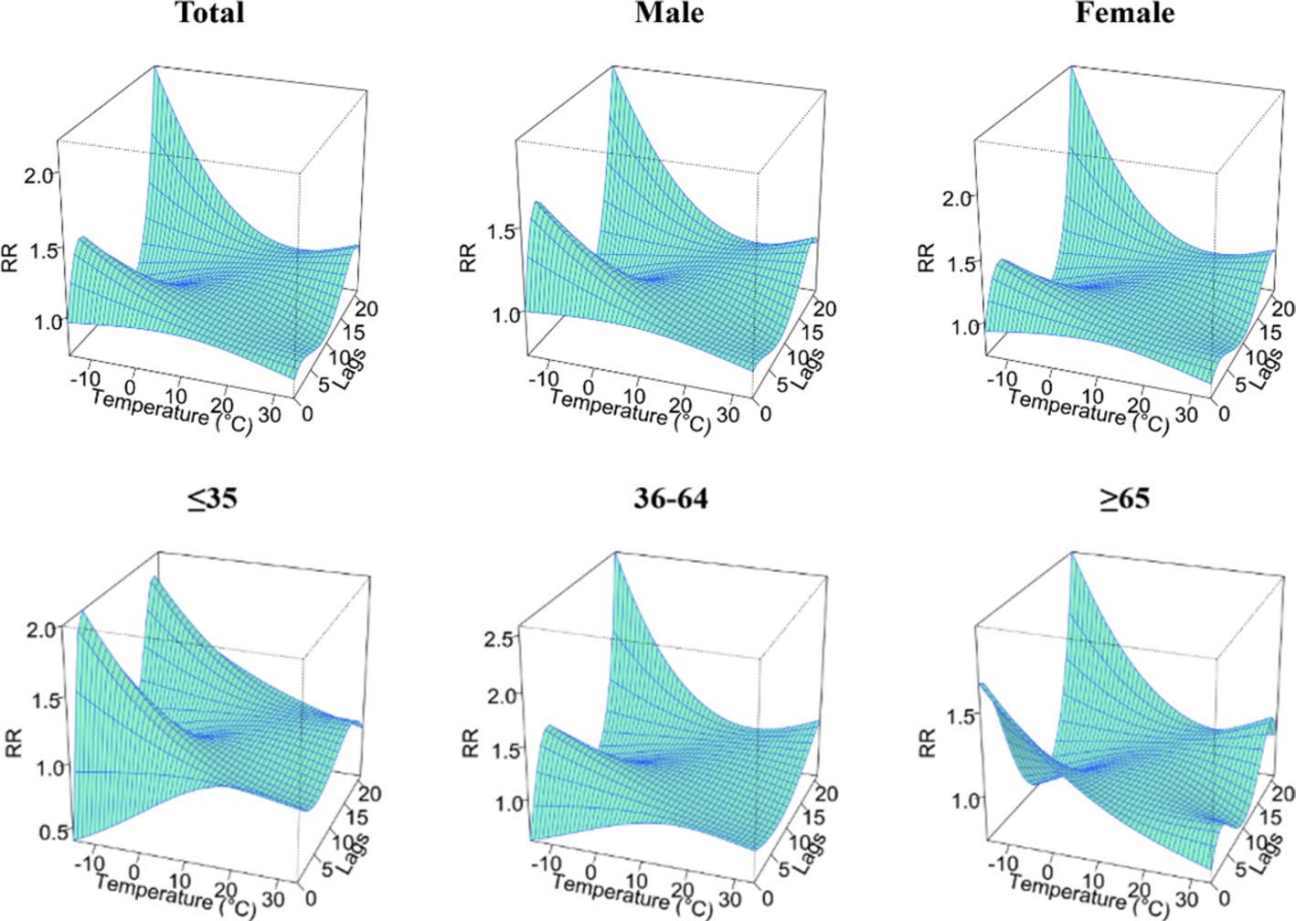
A three-dimensional graph of temperature hysteresis effects stratified by gender and age.

According to the DLNM analysis, the temperature effect of TB incidence in Yingjisha County from January 2016 to June 2023 was nonlinearly related to the total population, gender and age stratification, as shown in Fig. 3. Taking the median average daily temperature (15.3 °C) in Yingjisha County from 2016 to 2023 as a reference, the highest RR values for all groups occurred at – 15 °C at lag 21 days. The highest RR values for the age group of ≤ 35 years old occurred at lag 4 days only. The highest RR (95% CI) was 2.20 (0.77–6.29) for the total population; 2.00 (0.68–5.82) for the male group; 2.40 (0.78–7.36) for the female group; 2.00 (1.13–3.57) for the age group of ≤ 35 years old. The highest RR (95% CI) was 2.58 (0.84–7.96) for the age group of 36–64 years old; and the highest RR (95% CI) was 1.99 (0.65–6.09) for the age group of ≥ 65 years old.

**Figure 4:**
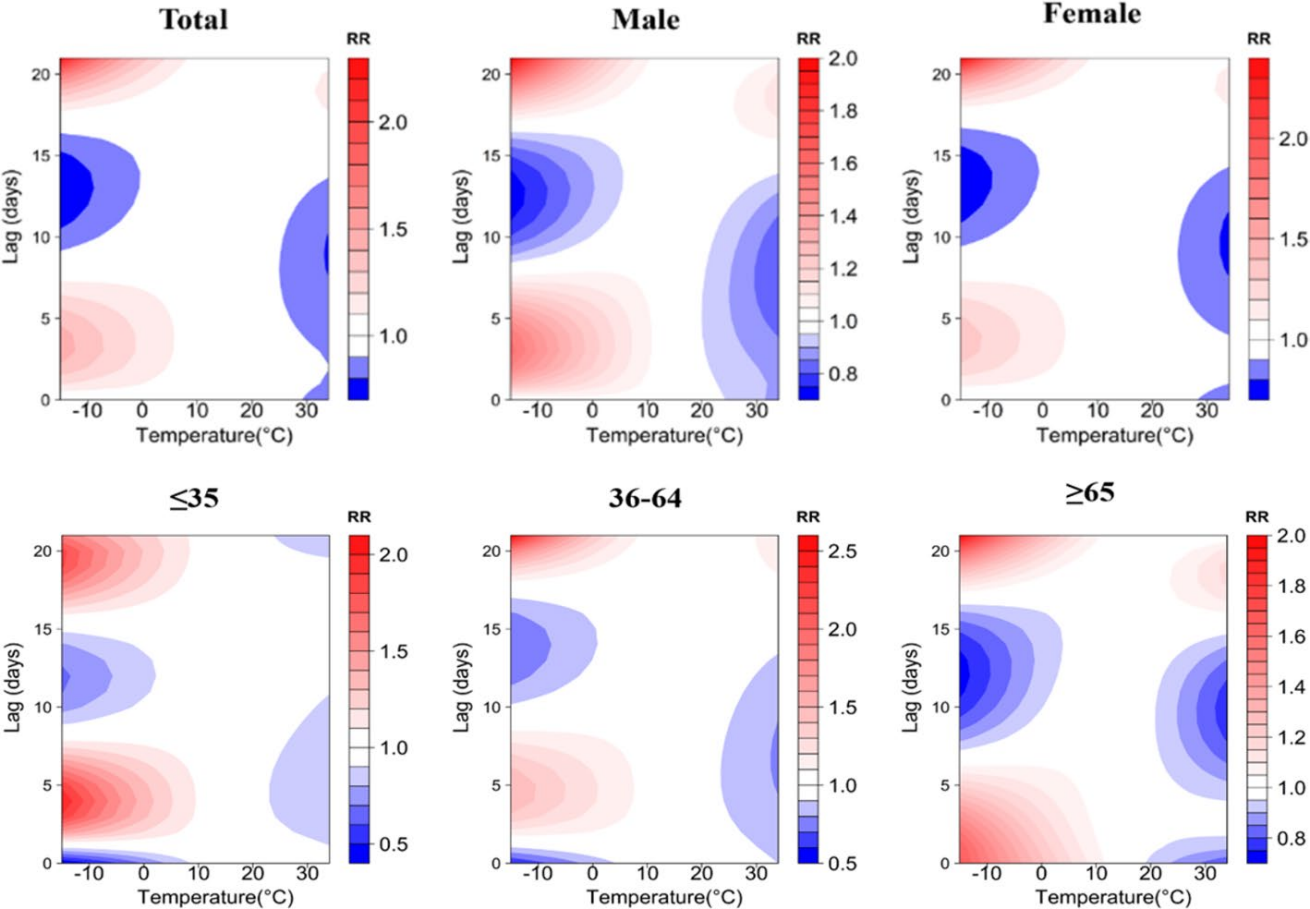
Contour plots of the effect of time lags and temperature on the TB incidence RR.

A contour plot of the effect of different temperatures and lag days on the RR value of TB episodes is demonstrated in Fig. 4, where the blue area indicates an RR less than 1 and the red area indicates an RR greater than 1. The bluer the area, the smaller the RR, indicating a lower risk of TB episodes. Using 15.3 °C (P_50_) as a reference, under low-temperature conditions, RR values for all groups were higher at lag 4 days and reached a maximum at lag 21 days. Only the risk of TB attack for those age group of ≥ 65 years old presented a hazard at lag 0 days. The risk of TB attack for those was lower at lag 0 days. The risk of TB attack for those age group of ≥ 65 years old presented a hazard at lag 0 days. Under high temperature conditions, the risk of TB incidence was low for the majority of the population, which would suggest that high temperature has a protective effect on the transmission of TB, but the risk of TB incidence increased at lag 18 days, with the exception of those age group of ≤ 35 years old.

**Figure 5:**
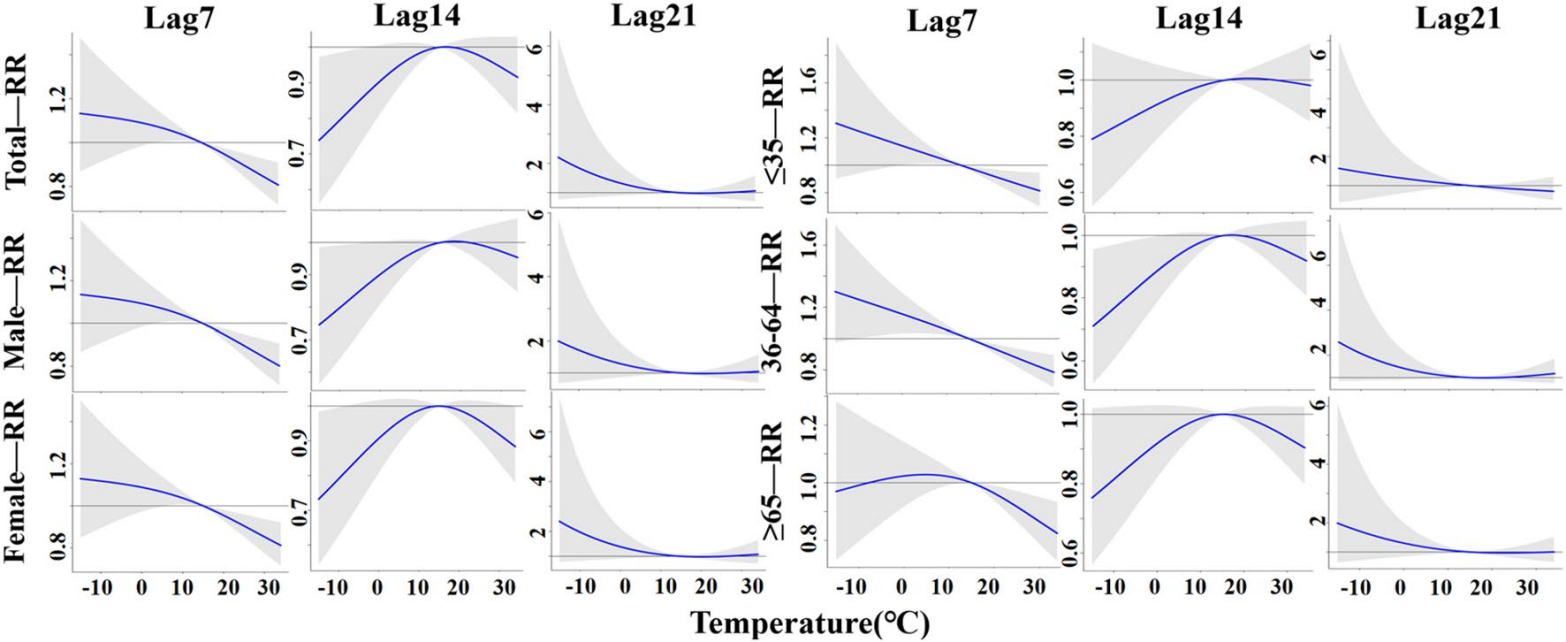
The lag-specific effect at different temperature on the TB incidence with reference at 15.3 °C.

In this study, 15.3 °C (P_50_) was set as the temperature threshold, and the effects of temperature at lag 7 days, lag 14 days and lag 21 days on the incidence rates of TB in the whole population, men, women, people ≤ 35 years of age, people 36–64 years of age, and people ≥ 65 years of age were analyzed in detail and are shown in Fig. 5. For each group, at lag 7 days and 21 days, low temperatures (< 15.3 °C) caused the increase in RR, especially at – 15 °C where RR values reached a maximum, and gradually decreased with increasing temperature; however, at lag 14 days, RR values also gradually increased with increasing temperature, and this phenomenon was also observed at lag 7 days for the age group of ≥ 65 years old population. High temperatures (> 15.3 °C) resulted in a decrease in RR and a steady decrease in RR values with increasing temperature, but the risk of developing TB increased with increasing temperature in all groups at lag 21 days, especially in the age group of 35–64 years old, which had a significant increase in risk.

In this paper, we investigated the cumulative lag effect of low temperature level (P_10_ = − 3.6 °C) versus high temperature level (P_90_ = 26.7 °C) on the incidence of TB at lag 0–21 days, which is shown in Tables 3, 4. In the table, the results stratified by gender and age are presented. At low temperature levels, the cumulative risk of TB incidence at lag 0–9 days basically increased with increasing lag days in all groups; at lag 10–17 days, it began to decrease gradually with increasing lag days; and at lag 18–21 days, it increased again, and the cumulative RR was the greatest at lag 21 days in all groups, except for the male population group and the age group of ≥ 65 years old population. At high temperature levels, the cumulative risk of TB incidence decreased with increasing lag days in all groups at lag 0–15 days; it tended to increase slightly at lag 16–21 days in all groups except the age group of ≤ 35 years old, and the cumulative RR was greatest at lag 0 days in all groups. In the gender stratification (Table 3), the male group had the highest RR for TB hypothermia levels, with a cumulative RR of 4.30 at lag 8 days and 4.22 at lag 21 days, and the female group had the lowest RR for TB hypothermia levels, with a cumulative RR of 3.08 at lag 21 days. When the temperature exceeded 15.3 °C, the protective effect of hypothermia levels on the incidence of TB increased with the lag period increased, with the greatest protective effect in the female group, with a cumulative RR of 0.27 at lag 21 days. In the age stratification (Table 4), the RR for low temperature levels of TB was highest in those age group of ≤ 35 years old, with a cumulative RR of 6.90 at lag 21 days, and lowest in those aged 36–64 years of age, with a cumulative RR of 3.18 at lag 21 days. When the temperature was above 15.3℃, the protective effect of high temperature levels on TB incidence increased with lag time, with the greatest protective effect in those age group of ≤ 35 years old, with a cumulative RR of 0.22 at lag 21 days.

**Table 3:** Cumulative RR estimates with 95% CI of the effect of different temperature on TB incidence (gender).

Lag	Low level (P_10_ = − 3.6 °C)			High level (P_90_ = 26.7 °C)		
	Total	Male	Female	Total	Male	Female
0	1.00 (0.55–1.82)	1.02 (0.55–1.87)	0.99 (0.52–1.87)	0.92 (0.69–1.22)	0.93 (0.70–1.24)	0.91 (0.67–1.23)
1	1.13 (0.49–2.64)	1.19 (0.50–2.81)	1.07 (0.43–2.66)	0.85 (0.55–1.31)	0.86 (0.56–1.34)	0.85 (0.54–1.35)
2	1.37 (0.56–3.37)	1.51 (0.61–3.76)	1.24 (0.47–3.27)	0.79 (0.48–1.30)	0.79 (0.48–1.31)	0.81 (0.48–1.37)
3	1.71 (0.71–4.09)	1.97 (0.81–4.77)	1.48 (0.58–3.80)	0.73 (0.44–1.23)	0.72 (0.42–1.22)	0.76 (0.44–1.32)
4	2.12 (0.91–4.93)	2.55 (1.09–5.96)	1.77 (0.71–4.41)	0.67 (0.39–1.14)	0.64 (0.37–1.10)	0.71 (0.40–1.24)
5	2.57 (1.12–5.93)	3.18 (1.37–7.37)	2.09 (0.85–5.17)	0.60 (0.35–1.03)	0.57 (0.33–0.98)	0.65 (0.36–1.15)
6	2.99 (1.29–6.93)	3.75 (1.61–8.75)	2.40 (0.97–5.97)	0.54 (0.31–0.93)	0.50 (0.28–0.88)	0.58 (0.32–1.04)
7	3.31 (1.42–7.70)	4.16 (1.77–9.74)	2.65 (1.06–6.61)	0.47 (0.27–0.83)	0.44 (0.24–0.78)	0.51 (0.28–0.94)
8	3.45 (1.48–8.06)	4.3 (1.83–10.12)	2.78 (1.11–6.97)	0.41 (0.23–0.74)	0.38 (0.21–0.70)	0.45 (0.24–0.83)
9	3.41 (1.44–8.04)	4.17 (1.75–9.93)	2.79 (1.10–7.05)	0.36 (0.20–0.67)	0.34 (0.18–0.64)	0.39 (0.20–0.74)
10	3.19 (1.32–7.76)	3.83 (1.56–9.41)	2.66 (1.02–6.93)	0.32 (0.17–0.61)	0.31 (0.16–0.59)	0.34 (0.17–0.67)
11	2.87 (1.13–7.32)	3.37 (1.30–8.72)	2.44 (0.89–6.68)	0.29 (0.15–0.56)	0.28 (0.14–0.56)	0.30 (0.15–0.61)
12	2.50 (0.93–6.76)	2.89 (1.05–7.94)	2.16 (0.74–6.28)	0.26 (0.13–0.53)	0.26 (0.13–0.54)	0.27 (0.13–0.56)
13	2.15 (0.76–6.11)	2.46 (0.85–7.12)	1.87 (0.61–5.74)	0.25 (0.12–0.51)	0.25 (0.12–0.54)	0.25 (0.11–0.53)
14	1.85 (0.63–5.44)	2.11 (0.70–6.34)	1.62 (0.51–5.12)	0.24 (0.11–0.51)	0.25 (0.11–0.54)	0.23 (0.10–0.52)
15	1.63 (0.55–4.88)	1.88 (0.61–5.73)	1.41 (0.44–4.55)	0.23 (0.11–0.51)	0.25 (0.11–0.56)	0.23 (0.10–0.51)
16	1.50 (0.49–4.56)	1.75 (0.56–5.46)	1.27 (0.39–4.19)	0.24 (0.11–0.53)	0.26 (0.11–0.59)	0.23 (0.10–0.53)
17	1.46 (0.46–4.60)	1.75 (0.54–5.63)	1.22 (0.36–4.14)	0.25 (0.11–0.56)	0.27 (0.12–0.63)	0.23 (0.10–0.55)
18	1.55 (0.47–5.06)	1.89 (0.56–6.35)	1.26 (0.36–4.48)	0.26 (0.11–0.61)	0.29 (0.12–0.69)	0.25 (0.10–0.60)
19	1.82 (0.55–5.96)	2.24 (0.66–7.59)	1.46 (0.41–5.20)	0.28 (0.12–0.66)	0.30 (0.12–0.75)	0.26 (0.10–0.65)
20	2.40 (0.78–7.35)	2.93 (0.93–9.26)	1.95 (0.59–6.39)	0.29 (0.12–0.70)	0.32 (0.13–0.79)	0.27 (0.11–0.70)
21	3.64 (1.22–10.8)	4.22 (1.37–13.03)	3.08 (0.97–9.72)	0.29 (0.11–0.75)	0.32 (0.12–0.85)	0.27 (0.10–0.75)

**Table 4:** Cumulative RR estimates with 95% CI of the effect of different temperature on TB incidence (age).

Lag	Low level (P10 = − 3.6 °)			High level (P90 = 26.7 °C)		
	0–35	36–64	65–100	0–35	36–64	65–100
0	0.62 (0.27–1.38)	0.77 (0.40–1.47)	1.36 (0.73–2.54)	1.02 (0.72–1.45)	0.96 (0.70–1.32)	0.87 (0.64–1.17)
1	0.59 (0.19–1.88)	0.74 (0.29–1.86)	1.80 (0.75–4.34)	0.97 (0.57–1.64)	0.89 (0.55–1.43)	0.81 (0.51–1.27)
2	0.74 (0.22–2.57)	0.82 (0.31–2.22)	2.30 (0.90–5.84)	0.88 (0.48–1.60)	0.80 (0.47–1.38)	0.77 (0.46–1.31)
3	1.08 (0.32–3.61)	1.01 (0.38–2.66)	2.81 (1.14–6.93)	0.77 (0.41–1.44)	0.71 (0.40–1.25)	0.75 (0.43–1.30)
4	1.61 (0.50–5.19)	1.29 (0.50–3.31)	3.29 (1.38–7.83)	0.66 (0.35–1.25)	0.62 (0.35–1.10)	0.72 (0.41–1.27)
5	2.33 (0.74–7.36)	1.66 (0.65–4.21)	3.67 (1.56–8.65)	0.57 (0.30–1.08)	0.53 (0.30–0.96)	0.68 (0.38–1.21)
6	3.09 (0.98–9.77)	2.07 (0.81–5.31)	3.90 (1.64–9.25)	0.49 (0.26–0.95)	0.46 (0.25–0.83)	0.62 (0.35–1.12)
7	3.68 (1.17–11.65)	2.48 (0.96–6.38)	3.95 (1.65–9.43)	0.43 (0.22–0.85)	0.39 (0.21–0.73)	0.56 (0.31–1.02)
8	3.91 (1.24–12.36)	2.79 (1.08–7.21)	3.83 (1.60–9.19)	0.39 (0.19–0.78)	0.34 (0.18–0.65)	0.49 (0.26–0.92)
9	3.75 (1.18–11.93)	2.93 (1.12–7.67)	3.57 (1.47–8.68)	0.36 (0.17–0.74)	0.30 (0.15–0.58)	0.43 (0.23–0.82)
10	3.30 (1.00–10.87)	2.89 (1.07–7.80)	3.23 (1.29–8.10)	0.34 (0.16–0.72)	0.27 (0.13–0.54)	0.37 (0.19–0.73)
11	2.76 (0.79–9.67)	2.68 (0.94–7.63)	2.85 (1.08–7.54)	0.32 (0.14–0.72)	0.24 (0.12–0.51)	0.33 (0.16–0.67)
12	2.27 (0.60–8.57)	2.37 (0.78–7.18)	2.48 (0.88–6.98)	0.31 (0.13–0.73)	0.22 (0.10–0.49)	0.29 (0.14–0.62)
13	1.90 (0.47–7.68)	2.03 (0.63–6.48)	2.16 (0.73–6.39)	0.31 (0.13–0.75)	0.21 (0.09–0.48)	0.27 (0.12–0.59)
14	1.68 (0.40–7.10)	1.71 (0.51–5.68)	1.89 (0.62–5.78)	0.31 (0.12–0.78)	0.21 (0.09–0.47)	0.26 (0.12–0.58)
15	1.61 (0.37–6.97)	1.45 (0.43–4.95)	1.69 (0.54–5.28)	0.31 (0.12–0.80)	0.20 (0.09–0.48)	0.26 (0.11–0.58)
16	1.71 (0.39–7.53)	1.28 (0.37–4.46)	1.58 (0.50–5.00)	0.31 (0.12–0.81)	0.20 (0.08–0.49)	0.26 (0.11–0.61)
17	2.02 (0.44–9.19)	1.21 (0.33–4.35)	1.55 (0.47–5.08)	0.30 (0.11–0.82)	0.21 (0.09–0.51)	0.28 (0.12–0.67)
18	2.63 (0.56–12.48)	1.24 (0.33–4.68)	1.63 (0.48–5.57)	0.29 (0.11–0.81)	0.22 (0.09–0.55)	0.30 (0.12–0.74)
19	3.67 (0.77–17.47)	1.45 (0.38–5.49)	1.87 (0.55–6.40)	0.28 (0.10–0.78)	0.23 (0.09–0.59)	0.33 (0.13–0.82)
20	5.20 (1.18–22.81)	1.96 (0.55–6.97)	2.39 (0.76–7.51)	0.25 (0.09–0.72)	0.24 (0.09–0.65)	0.34 (0.13–0.88)
21	6.90 (1.61–29.49)	3.18 (0.91–11.10)	3.45 (1.15–10.39)	0.22 (0.07–0.68)	0.26 (0.09–0.74)	0.33 (0.12–0.92)

#### Effect of RH on the development of TB

**Figure 6:**
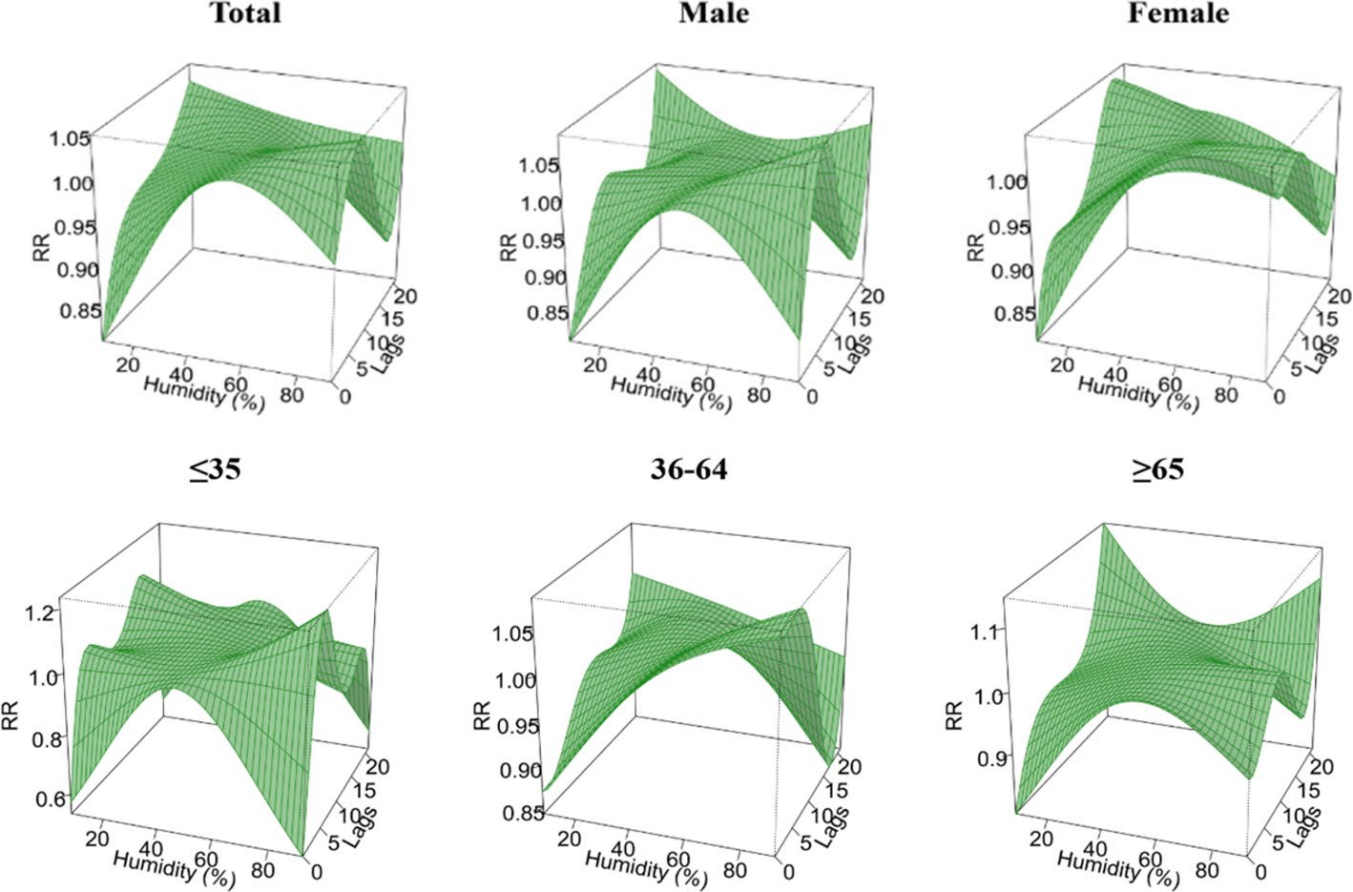
A three-dimensional diagram of the hysteresis effect of different RH levels by gender and age.

Using the median daily average relative humidity (43%) from 2016 to 2023 in Yingjisha County as a reference, the highest RR values for all groups appeared at 92% relative humidity, and only for the female group and the group of people ≥ 65 years old at 70% and 8% relative humidity. The highest RR (95% CI) for the total population appeared at 1.05 (0.92–1.19) at lag 6 days; the highest RR (95% CI) for the male group appeared at 1.08 (0.95–1.23) at lag 6 days; the highest RR (95% CI) for the female group appeared at 1.05 (0.88–1.25) at lag 0 days; the highest RR (95% CI) in the ≤ 35 age group occurred at lag 4 days and was 1.24 (1.02–1.50), the highest RR (95% CI) in the 36–64 age group occurred at lag 5 days and was 1.08 (0.93–1.26), the highest RR (95% CI) in the ≥ 65 age group occurred at lag 21 days and was 1.15 (0.83–1.59), as shown in Fig. 6.

**Figure 7:**
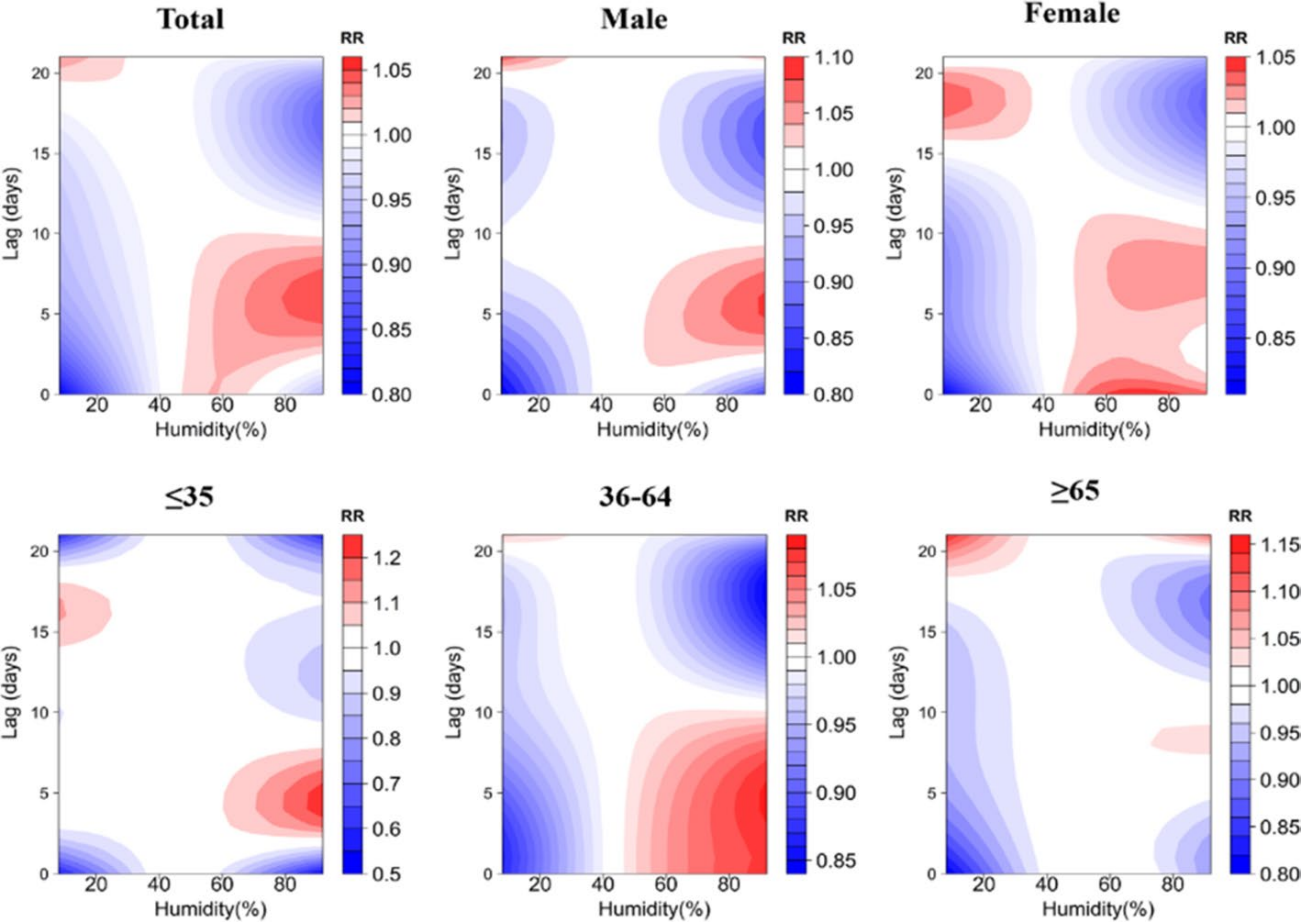
Contour plots of the effect of time lags and RH on the TB incidence RR.

Figure 7 demonstrates contour plots of the effect of different relative humidity and number of lag days on the RR value of TB incidence. Using 43% (P_50_) as a reference, under low RH conditions, the RR values of the groups were higher after lag 15 days and reached a maximum at lag 21 days, only the risk of TB incidence in people ≤ 35 years old showed a low risk at lag 21 days, and the RR value of the female group reached a maximum at lag 18 days. Under high relative humidity conditions, the RR values of all groups were higher at lag 5 days, and for the majority of the population, the risk of TB incidence was low at lag 15 days, but it is important to note that the risk of TB incidence in the ≥ 65 years of age increased at lag 21 days, as shown in Fig. 7.

**Figure 8:**
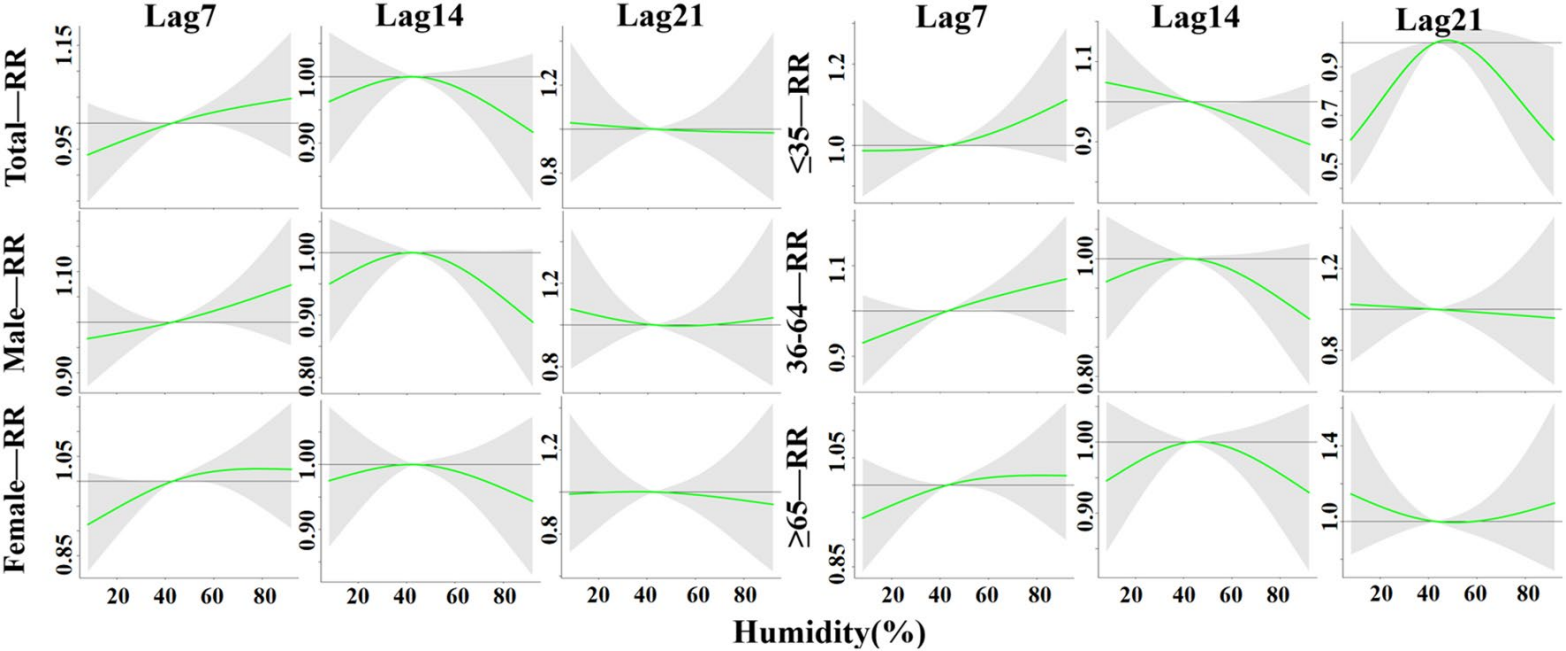
The lag-specific effect at different RH on the TB incidence with reference at 43%.

In this paper, we set 43% (P_50_) as the RH threshold and analyzed in detail the effects of RH at lag 7 days, lag 14 days and lag 21 days on the incidence rates of TB in the whole population, males, females, ≤ 35 years olds, 36–64 years olds, and ≥ 65 years olds, as shown in Fig. 8. For each group, at lag 7 days and lag 14 days, low RH (< 43%) was the protective factor, with RR values gradually increasing with increasing relative humidity, with the exception of the age group of ≤ 35 years old at lag 14 days; at lag 21 days, RR values were gradually decreasing with increasing relative humidity, with only the age group of ≤ 35 years old at lag 21 days being consistent with the rest of the groups at lag 7 days and lag 14 days. High relative humidity (> 43%) led to an increase in RR in all groups at lag 7 days; RR values steadily decreased with increasing temperature in all groups at lag 14 days, in agreement with the female and ≥ 36 year old groups at lag 7 days; the risk of TB decreased with increasing temperature in all groups at lag 21 days, but increased in the male and ≥ 65 years old groups, with a significant increase in the risk in the ≥ 65 years group.

In this paper, we investigated the cumulative lag effect of low RH levels (P_10_ = 26%) versus high RH levels (P_90_ = 68%) on TB incidence at lag 0–21 days, which is shown in Tables 5, 6. In the table, the results stratified by gender and age are presented. At low RH levels, the cumulative risk of TB incidence for all groups at lag 0–19 days essentially decreased with increasing lag days; at lag 20–21 days, it began to increase slightly with increasing lag days, and began to increase steadily only for those ≤ 35 years of age at lag 13 days; the cumulative RR was greatest at lag 0 days for all groups. At high RH levels, the cumulative risk of TB incidence increased with lag days for all groups at lag 0–11 days; a decreasing trend was observed at lag 12–21 days, with all groups having the greatest cumulative RR at lag 9–11 days. In the gender stratification (Table 5), the highest RR value at low RH levels was equal for all groups, occurring at 0.92 at lag 0 days. When RH exceeded 43%, the protective effect of high RH levels on TB incidence increased with lag, with the male group having the greatest protective effect, with a cumulative RR of 0.90 at lag 21 days. In the age stratification (Table 6), the cumulative risk of TB incidence at low RH levels for the 36–64 years old had the highest RR for low RH levels of TB, with a cumulative RR of 0.94 at lag 0 days, and those ≤ 35 years old had the lowest RR for low temperature levels of TB, with a cumulative RR of 0.83 at lag 0 days. When RH exceeded 43%, the protective effect of high RH levels on TB incidence tended to decrease and then increase with lag, with ≤ 35 years old having the greatest protective effect, and the protective effect of the lag being greatest in the male group, with the cumulative RR of 0.90 at lag 21 days. The protective effect was greatest in the population, with a cumulative RR of 0.63 at lag 21 days.

**Table 5:** Cumulative RR estimates with 95% CI of the effect of different RH on TB incidence (gender).

Lag	Low level (P_10_ = 26%)			High level (P_90_ = 68%)		
	Total	Male	Female	Total	Male	Female
0	0.92 (0.80–1.05)	0.92 (0.81–1.06)	0.92 (0.79–1.06)	1.01 (0.87–1.17)	0.97 (0.83–1.13)	1.05 (0.89–1.22)
1	0.86 (0.70–1.05)	0.86 (0.70–1.05)	0.86 (0.69–1.07)	1.02 (0.82–1.28)	0.97 (0.78–1.23)	1.08 (0.85–1.36)
2	0.81 (0.64–1.02)	0.81 (0.64–1.02)	0.82 (0.64–1.05)	1.05 (0.81–1.35)	1.00 (0.77–1.29)	1.10 (0.84–1.44)
3	0.77 (0.60–0.99)	0.76 (0.60–0.98)	0.78 (0.60–1.02)	1.07 (0.83–1.39)	1.03 (0.79–1.35)	1.12 (0.85–1.48)
4	0.74 (0.58–0.96)	0.73 (0.57–0.95)	0.75 (0.57–0.99)	1.10 (0.85–1.44)	1.07 (0.82–1.41)	1.14 (0.86–1.51)
5	0.72 (0.55–0.93)	0.71 (0.54–0.93)	0.72 (0.55–0.96)	1.14 (0.87–1.49)	1.12 (0.85–1.48)	1.16 (0.87–1.55)
6	0.69 (0.53–0.91)	0.69 (0.52–0.91)	0.70 (0.52–0.93)	1.17 (0.89–1.54)	1.17 (0.88–1.54)	1.19 (0.88–1.59)
7	0.68 (0.51–0.90)	0.68 (0.51–0.91)	0.67 (0.50–0.91)	1.21 (0.91–1.60)	1.21 (0.91–1.60)	1.21 (0.90–1.64)
8	0.66 (0.49–0.88)	0.67 (0.5–0.90)	0.65 (0.47–0.88)	1.24 (0.93–1.64)	1.24 (0.92–1.65)	1.24 (0.92–1.69)
9	0.64 (0.48–0.87)	0.67 (0.49–0.91)	0.62 (0.45–0.86)	1.26 (0.94–1.69)	1.25 (0.93–1.69)	1.27 (0.93–1.74)
10	0.63 (0.46–0.86)	0.66 (0.48–0.91)	0.60 (0.43–0.84)	1.27 (0.94–1.72)	1.26 (0.92–1.71)	1.29 (0.93–1.79)
11	0.62 (0.45–0.86)	0.66 (0.47–0.92)	0.58 (0.41–0.83)	1.27 (0.93–1.75)	1.24 (0.90–1.72)	1.31 (0.93–1.84)
12	0.61 (0.43–0.85)	0.65 (0.46–0.92)	0.57 (0.40–0.82)	1.26 (0.90–1.76)	1.22 (0.87–1.71)	1.31 (0.92–1.87)
13	0.60 (0.42–0.85)	0.64 (0.45–0.92)	0.56 (0.38–0.82)	1.24 (0.87–1.75)	1.18 (0.83–1.69)	1.30 (0.90–1.88)
14	0.59 (0.41–0.85)	0.63 (0.44–0.92)	0.56 (0.38–0.82)	1.20 (0.84–1.72)	1.13 (0.78–1.64)	1.28 (0.87–1.87)
15	0.59 (0.40–0.86)	0.62 (0.43–0.91)	0.56 (0.37–0.83)	1.16 (0.80–1.68)	1.08 (0.74–1.59)	1.24 (0.83–1.83)
16	0.59 (0.40–0.86)	0.61 (0.41–0.91)	0.56 (0.38–0.85)	1.11 (0.75–1.62)	1.03 (0.70–1.53)	1.19 (0.79–1.78)
17	0.59 (0.40–0.87)	0.60 (0.40–0.90)	0.57 (0.38–0.87)	1.05 (0.71–1.57)	0.98 (0.65–1.48)	1.13 (0.74–1.72)
18	0.59 (0.39–0.88)	0.59 (0.39–0.90)	0.59 (0.38–0.90)	1.00 (0.66–1.52)	0.94 (0.62–1.45)	1.07 (0.69–1.66)
19	0.59 (0.39–0.90)	0.59 (0.39–0.91)	0.60 (0.39–0.93)	0.96 (0.63–1.47)	0.91 (0.59–1.42)	1.01 (0.64–1.59)
20	0.60 (0.39–0.92)	0.60 (0.38–0.92)	0.61 (0.39–0.96)	0.93 (0.60–1.44)	0.90 (0.57–1.40)	0.97 (0.61–1.54)
21	0.61 (0.39–0.95)	0.61 (0.39–0.97)	0.61 (0.38–0.98)	0.92 (0.58–1.45)	0.90 (0.56–1.43)	0.95 (0.58–1.54)

**Table 6:** Cumulative RR estimates with 95% CI of the effect of different RH on TB incidence (age).

Lag	Low level (P_10_ = 26%)			High level (P_90_ = 68%)		
	0–35	36–64	65–100	0–35	36–64	65–100
0	0.83 (0.70–0.97)	0.94 (0.82–1.08)	0.92 (0.79–1.06)	0.85 (0.69–1.03)	1.05 (0.90–1.23)	1.00 (0.86–1.17)
1	0.74 (0.58–0.95)	0.88 (0.71–1.09)	0.86 (0.69–1.07)	0.80 (0.59–1.09)	1.11 (0.87–1.40)	1.00 (0.79–1.26)
2	0.71 (0.53–0.94)	0.83 (0.65–1.06)	0.81 (0.63–1.04)	0.82 (0.58–1.16)	1.16 (0.89–1.52)	0.99 (0.76–1.29)
3	0.69 (0.51–0.93)	0.79 (0.61–1.02)	0.78 (0.60–1.01)	0.88 (0.62–1.26)	1.22 (0.92–1.62)	0.99 (0.75–1.30)
4	0.69 (0.50–0.94)	0.75 (0.57–0.98)	0.75 (0.57–0.98)	0.96 (0.67–1.37)	1.29 (0.97–1.71)	0.99 (0.75–1.31)
5	0.68 (0.50–0.94)	0.71 (0.54–0.94)	0.72 (0.55–0.96)	1.04 (0.73–1.48)	1.35 (1.01–1.80)	0.99 (0.75–1.32)
6	0.68 (0.49–0.95)	0.69 (0.51–0.92)	0.70 (0.52–0.95)	1.11 (0.77–1.59)	1.41 (1.05–1.89)	1.01 (0.75–1.35)
7	0.67 (0.48–0.94)	0.66 (0.49–0.90)	0.69 (0.51–0.93)	1.16 (0.81–1.67)	1.46 (1.09–1.97)	1.02 (0.76–1.38)
8	0.66 (0.47–0.94)	0.65 (0.47–0.88)	0.67 (0.49–0.92)	1.18 (0.82–1.71)	1.51 (1.11–2.05)	1.04 (0.77–1.42)
9	0.65 (0.45–0.93)	0.63 (0.46–0.87)	0.65 (0.47–0.90)	1.18 (0.81–1.72)	1.54 (1.13–2.11)	1.06 (0.77–1.45)
10	0.64 (0.44–0.93)	0.62 (0.44–0.86)	0.64 (0.45–0.89)	1.15 (0.78–1.70)	1.56 (1.13–2.16)	1.08 (0.78–1.49)
11	0.63 (0.43–0.94)	0.61 (0.43–0.86)	0.62 (0.44–0.88)	1.10 (0.73–1.66)	1.56 (1.11–2.19)	1.08 (0.77–1.53)
12	0.63 (0.42–0.95)	0.60 (0.42–0.86)	0.61 (0.42–0.88)	1.05 (0.68–1.61)	1.54 (1.08–2.20)	1.08 (0.76–1.55)
13	0.64 (0.41–0.98)	0.60 (0.41–0.87)	0.59 (0.41–0.87)	0.99 (0.63–1.55)	1.50 (1.04–2.18)	1.07 (0.74–1.55)
14	0.65 (0.42–1.02)	0.59 (0.40–0.87)	0.58 (0.39–0.87)	0.94 (0.59–1.50)	1.45 (0.98–2.14)	1.05 (0.72–1.54)
15	0.68 (0.43–1.07)	0.58 (0.39–0.87)	0.57 (0.38–0.86)	0.90 (0.56–1.45)	1.38 (0.92–2.06)	1.02 (0.69–1.51)
16	0.71 (0.44–1.13)	0.58 (0.39–0.87)	0.57 (0.38–0.86)	0.86 (0.53–1.41)	1.31 (0.86–1.98)	0.98 (0.65–1.47)
17	0.74 (0.46–1.20)	0.57 (0.38–0.87)	0.57 (0.37–0.87)	0.83 (0.50–1.38)	1.23 (0.80–1.89)	0.94 (0.61–1.43)
18	0.76 (0.46–1.25)	0.57 (0.37–0.88)	0.57 (0.37–0.89)	0.81 (0.48–1.36)	1.15 (0.73–1.82)	0.90 (0.58–1.39)
19	0.75 (0.45–1.24)	0.57 (0.37–0.89)	0.59 (0.37–0.92)	0.77 (0.45–1.31)	1.09 (0.68–1.74)	0.87 (0.55–1.36)
20	0.70 (0.42–1.16)	0.57 (0.36–0.90)	0.61 (0.38–0.96)	0.71 (0.42–1.22)	1.04 (0.65–1.68)	0.85 (0.54–1.35)
21	0.58 (0.34–1.00)	0.58 (0.36–0.93)	0.64 (0.39–1.03)	0.63 (0.36–1.11)	1.02 (0.62–1.68)	0.87 (0.54–1.40)

#### The effect of WS on the development of TB

**Figure 9:**
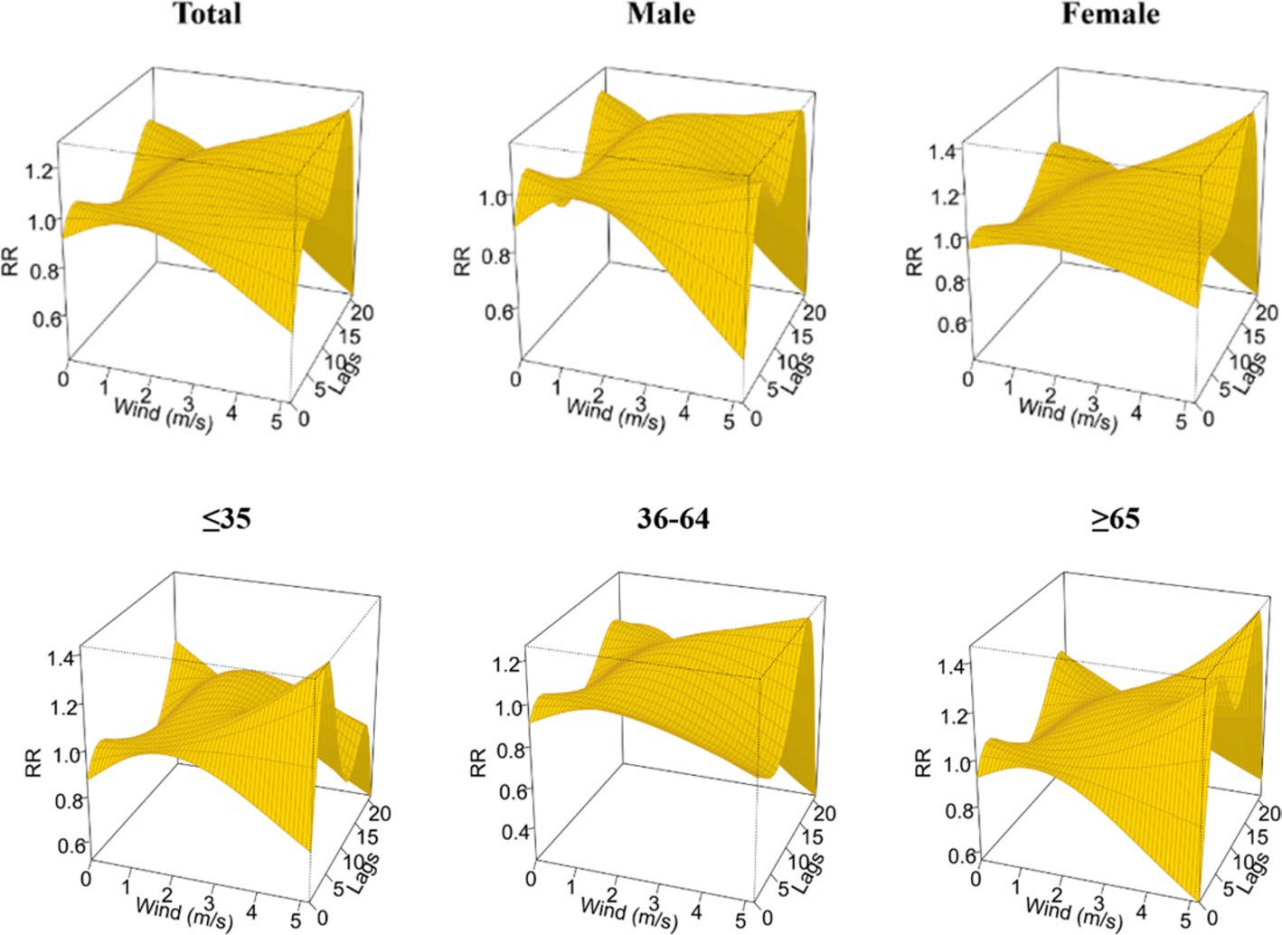
A three-dimensional graph of the lag effect of different WS stratified by gender and age.

Using the median daily average wind speed (0.8 m/s) from 2016 to 2023 in Yingjisha County as a reference, the highest RR value for each group appeared at 5.2 m/s. The highest RR (95% CI) for the total population appeared at 1.30 (0.78–2.16) at lag 16 days; the highest RR (95% CI) for the male group appeared at 1.17 (0.69–1.98) at lag 16 days; the highest RR (95% CI) for the female group appeared at 1.43 (0.86–2.35) at lag 15 days; the highest RR (95% CI) in the ≤ 35 age group occurred at lag 4 days and was 1.43 (0.78–2.65); the highest RR (95% CI) in the 36–64 age group occurred at lag 15 days and was 1.27 (0.78–2.07); the highest RR (95% CI) in the ≥ 65 age group occurred at lag 17 days and was 1.47 (0.81–2.67), as shown in Fig. 9.

**Figure 10:**
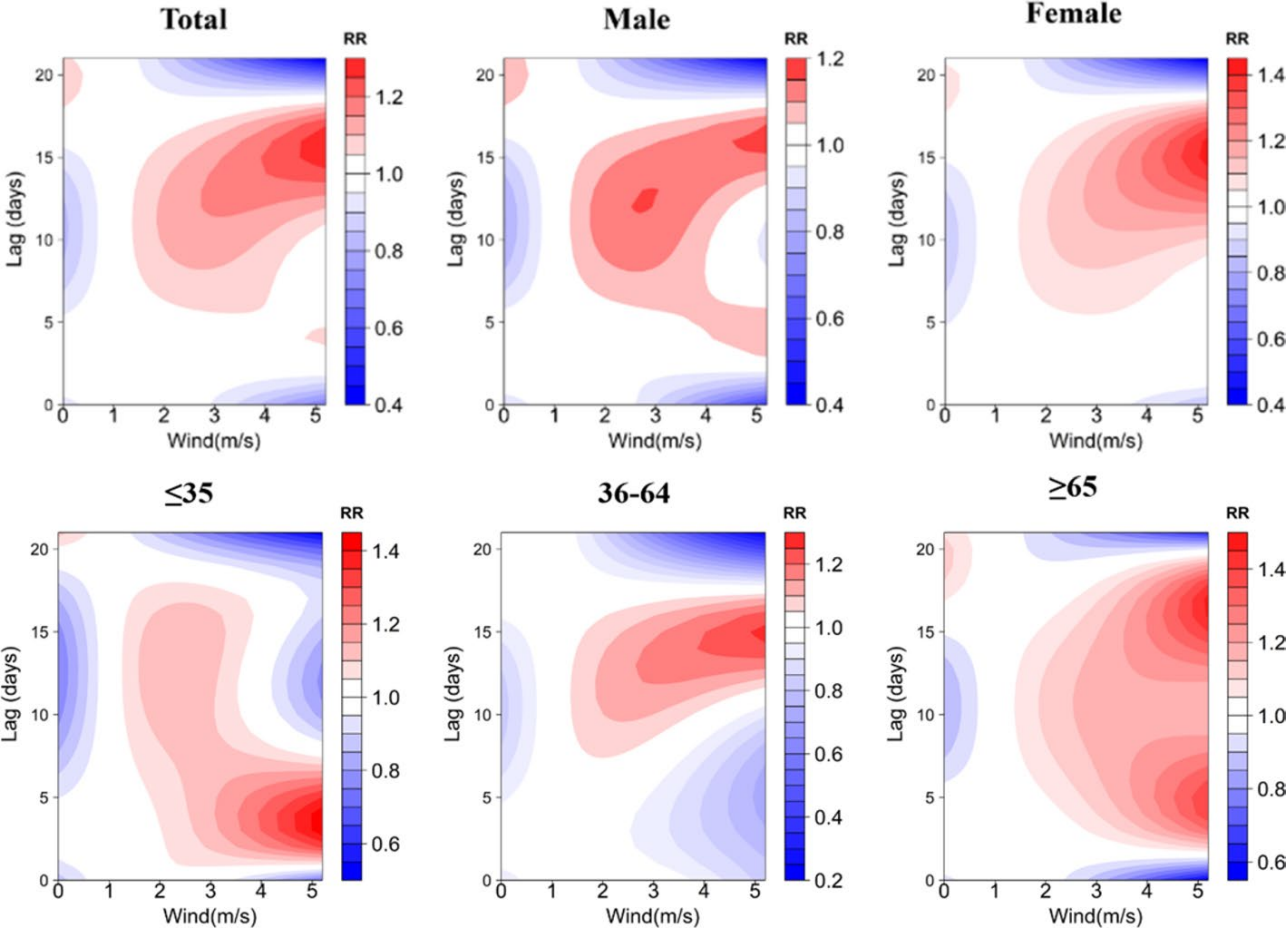
Contour plots of the effect of time lags and WS on the TB incidence RR.

Figure 10 demonstrates contour plots of the effect of different wind speeds and lag days on the RR values of TB incidence. Using 0.8 m/s (P_50_) as a reference, under low wind speed conditions, the RR values of the groups reached a maximum at lag 20 days, with the exception of the 36–64 year olds only. Under high wind speed conditions, the risk of developing TB was dangerously high at lag 15 days for all groups, with the exception of the 35 year olds, and at lag 3 days, as shown in Fig. 10.

**Figure 11:**
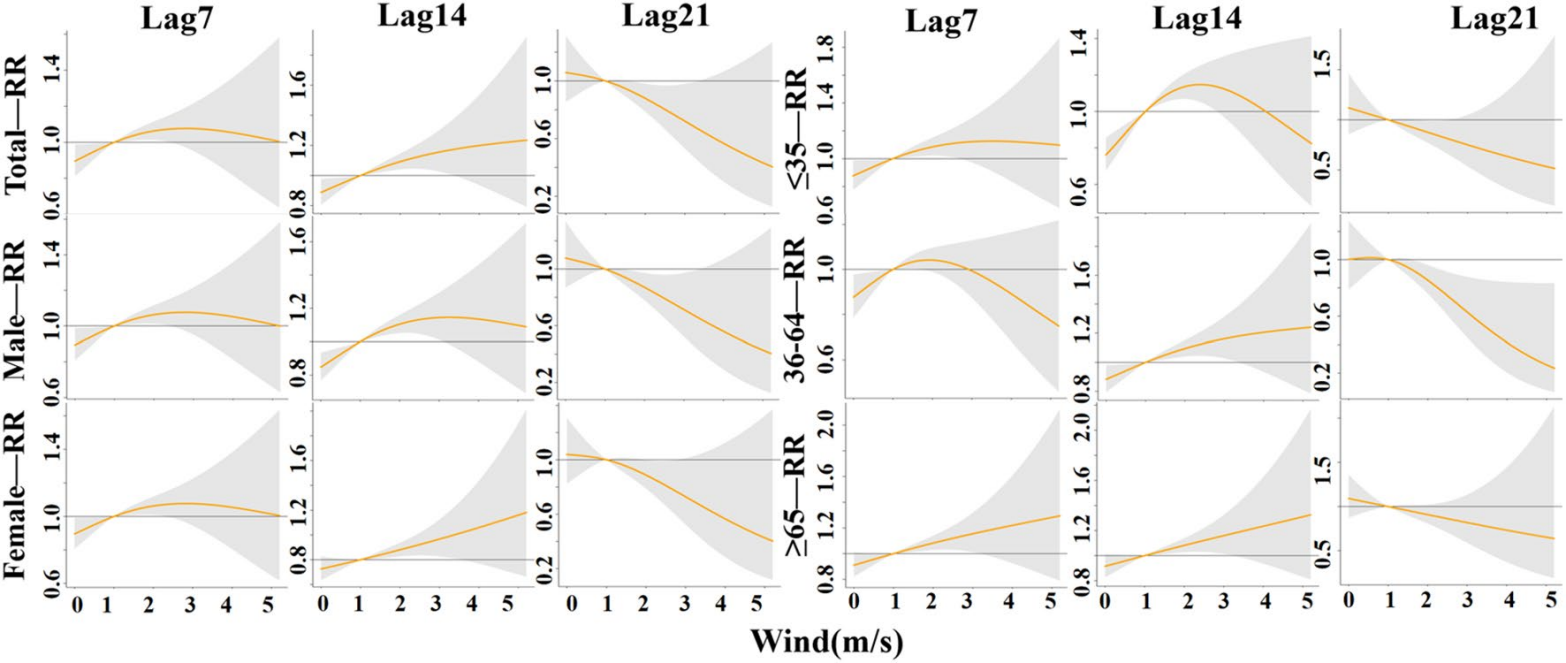
The lag-specifc effect at different WS on the TB incidence with reference at 0.8 m/s.

In this paper, we set 0.8 m/s (P_50_) as the wind speed threshold and analyzed in detail the effect of wind speed on the incidence of TB in the whole population, men, women, people ≤ 35 years old, people aged 36–64 years old, and people aged ≥ 65 years old at lag 7 days, lag 14 days, and lag 21 days, as shown in Fig. 11. For each group, at lag 7 days and 14 days, low wind speeds (< 0.8 m/s) reported lower RR values, and for all groups except those aged 35–64 years, the RR values reached a minimum at 0 m/s, and gradually increased with increasing wind speed; at lag 21 days, the RR values showed a gradual decrease with increasing wind speed. High wind speed (> 0.8 m/s) showed a trend of increasing and then decreasing TB risk at lag 7 days and 14 days; at lag 21 days, the risk of TB for all groups decreased with increasing wind speed.

In this paper, we investigated the cumulative lag effect of low wind speed level (P_10_ = 0.4 m/s) and high wind speed level (P_90_ = 1.6 m/s) on the incidence of TB at lag 0–21 days, as shown in Tables 7, 8. In the table, the results stratified by gender and age are presented. At low wind speed levels, the cumulative risk of TB incidence basically decreased with increasing lag days for all groups, except for the all-people, the male group, and the ≥ 64 years old, which showed an increasing trend at lag 0–4 days; thus, the cumulative RR was greatest at lag 0 days for all groups except for the all people, the male group, and the ≥ 64 years old. At high wind speed levels, the cumulative risk of TB incidence in all groups basically increased with increasing lag days; there was a slight decreasing trend at lag 16–21 days, and all groups had the greatest cumulative RR at lag 17 days, with only those ≤ 35 years old having the greatest RR at lag 19 days. In the gender stratification (Table 7), the male group had the highest RR for TB at low wind speed levels, with a cumulative RR of 0.99 at lag 4 days. When wind speed exceeded 0.8 m/s, the protective effect of high wind speed levels on the development of TB decreased with the lag period. In the age stratification (Table 8), the RR of low wind speed levels for TB was highest for those ≥ 65 years of age, with a cumulative RR of 0.98 at lag 2 days. The RR of low wind speed levels for TB was lowest for those ≤ 35 years of age, with a cumulative RR of 0.93 at lag 0 days. The protective effect of high wind speed levels against the development of TB decreased with lag when the wind speed exceeded 0.8 m/s. The protection of high wind speed levels against the onset of TB decreased with lag when wind speeds were higher than 0.8 m/s.

**Table 7:** Cumulative RR estimates with 95% CI of the effect of different WS on TB incidence (gender).

Lag	Low level (P_10_ = 0.4 m/s)			High level (P_90_ = 1.6 m/s)		
	Total	Male	Female	Total	Male	Female
0	0.95 (0.85–1.07)	0.94 (0.83–1.06)	0.97 (0.86–1.10)	1.02 (0.95–1.09)	1.02 (0.95–1.09)	1.01 (0.94–1.09)
1	0.95 (0.79–1.14)	0.93 (0.77–1.12)	0.96 (0.79–1.17)	1.01 (0.91–1.13)	1.01 (0.90–1.13)	1.02 (0.90–1.15)
2	0.95 (0.76–1.20)	0.95 (0.75–1.20)	0.96 (0.75–1.22)	1.01 (0.88–1.16)	1.00 (0.86–1.15)	1.02 (0.88–1.19)
3	0.96 (0.74–1.25)	0.97 (0.74–1.28)	0.95 (0.71–1.26)	1.00 (0.86–1.18)	0.98 (0.83–1.16)	1.03 (0.87–1.22)
4	0.96 (0.71–1.29)	0.99 (0.73–1.34)	0.93 (0.67–1.28)	1.01 (0.84–1.21)	0.98 (0.81–1.18)	1.04 (0.86–1.27)
5	0.94 (0.67–1.31)	0.98 (0.70–1.38)	0.90 (0.63–1.29)	1.03 (0.84–1.26)	0.99 (0.80–1.21)	1.07 (0.86–1.32)
6	0.90 (0.62–1.30)	0.94 (0.65–1.37)	0.86 (0.58–1.27)	1.06 (0.85–1.32)	1.01 (0.81–1.26)	1.10 (0.87–1.39)
7	0.84 (0.57–1.25)	0.89 (0.60–1.32)	0.81 (0.53–1.23)	1.10 (0.87–1.39)	1.06 (0.83–1.34)	1.15 (0.90–1.48)
8	0.78 (0.51–1.18)	0.81 (0.53–1.24)	0.75 (0.48–1.17)	1.17 (0.91–1.49)	1.12 (0.87–1.44)	1.21 (0.93–1.58)
9	0.71 (0.46–1.10)	0.73 (0.47–1.13)	0.69 (0.43–1.10)	1.24 (0.96–1.61)	1.20 (0.92–1.57)	1.28 (0.97–1.70)
10	0.64 (0.40–1.01)	0.64 (0.40–1.02)	0.63 (0.39–1.03)	1.34 (1.02–1.75)	1.31 (0.99–1.73)	1.37 (1.02–1.83)
11	0.58 (0.36–0.93)	0.56 (0.35–0.91)	0.58 (0.35–0.97)	1.44 (1.08–1.91)	1.42 (1.07–1.90)	1.45 (1.07–1.97)
12	0.52 (0.32–0.85)	0.50 (0.30–0.82)	0.54 (0.32–0.92)	1.55 (1.15–2.08)	1.55 (1.15–2.10)	1.55 (1.13–2.12)
13	0.48 (0.29–0.80)	0.44 (0.26–0.74)	0.51 (0.29–0.88)	1.65 (1.22–2.24)	1.68 (1.23–2.30)	1.63 (1.18–2.27)
14	0.45 (0.26–0.75)	0.40 (0.24–0.69)	0.48 (0.28–0.85)	1.75 (1.27–2.40)	1.80 (1.30–2.48)	1.71 (1.22–2.40)
15	0.43 (0.25–0.73)	0.38 (0.22–0.65)	0.47 (0.27–0.84)	1.82 (1.32–2.53)	1.89 (1.36–2.64)	1.77 (1.25–2.51)
16	0.42 (0.24–0.72)	0.36 (0.21–0.64)	0.47 (0.26–0.85)	1.87 (1.34–2.62)	1.96 (1.39–2.76)	1.81 (1.26–2.58)
17	0.42 (0.24–0.74)	0.36 (0.20–0.64)	0.48 (0.26–0.87)	1.89 (1.33–2.66)	1.97 (1.39–2.81)	1.81 (1.26–2.62)
18	0.43 (0.24–0.77)	0.37 (0.20–0.67)	0.49 (0.26–0.91)	1.86 (1.30–2.66)	1.95 (1.35–2.80)	1.79 (1.22–2.62)
19	0.45 (0.25–0.82)	0.39 (0.21–0.71)	0.51 (0.27–0.97)	1.80 (1.24–2.60)	1.87 (1.29–2.73)	1.74 (1.17–2.57)
20	0.47 (0.26–0.87)	0.41 (0.22–0.77)	0.53 (0.28–1.02)	1.71 (1.17–2.49)	1.77 (1.20–2.61)	1.66 (1.11–2.48)
21	0.49 (0.26–0.93)	0.43 (0.23–0.83)	0.55 (0.28–1.08)	1.59 (1.07–2.37)	1.64 (1.09–2.46)	1.56 (1.02–2.37)

**Table 8:** Cumulative RR estimates with 95% CI of the effect of different WS on TB incidence (age).

Lag	Low level (P_10_ = 0.4 m/s)			High level (P_90_ = 1.6 m/s)		
	0–35	36–64	65–100	0–35	36–64	65–100
0	0.93 (0.80–1.08)	0.95 (0.84–1.08)	0.96 (0.85–1.09)	1.04 (0.95–1.13)	1.03 (0.95–1.11)	1.00 (0.93–1.08)
1	0.90 (0.71–1.13)	0.93 (0.76–1.15)	0.96 (0.79–1.17)	1.07 (0.93–1.22)	1.03 (0.91–1.16)	0.99 (0.88–1.12)
2	0.88 (0.66–1.18)	0.93 (0.72–1.20)	0.98 (0.77–1.24)	1.09 (0.92–1.29)	1.02 (0.88–1.19)	0.99 (0.85–1.14)
3	0.87 (0.62–1.22)	0.93 (0.69–1.25)	1.00 (0.75–1.32)	1.12 (0.92–1.36)	1.01 (0.85–1.20)	0.99 (0.83–1.17)
4	0.86 (0.59–1.25)	0.91 (0.65–1.28)	1.00 (0.73–1.37)	1.15 (0.92–1.44)	1.01 (0.83–1.23)	1.00 (0.82–1.21)
5	0.83 (0.55–1.26)	0.88 (0.61–1.28)	0.99 (0.70–1.40)	1.19 (0.93–1.53)	1.01 (0.81–1.26)	1.02 (0.82–1.26)
6	0.79 (0.50–1.24)	0.84 (0.56–1.26)	0.96 (0.66–1.40)	1.25 (0.95–1.64)	1.04 (0.82–1.32)	1.06 (0.84–1.33)
7	0.73 (0.45–1.19)	0.78 (0.50–1.21)	0.91 (0.60–1.36)	1.32 (0.98–1.77)	1.08 (0.83–1.39)	1.11 (0.87–1.42)
8	0.66 (0.39–1.11)	0.71 (0.45–1.13)	0.84 (0.55–1.30)	1.41 (1.03–1.92)	1.13 (0.86–1.48)	1.18 (0.91–1.53)
9	0.59 (0.34–1.01)	0.64 (0.40–1.05)	0.77 (0.49–1.21)	1.52 (1.09–2.11)	1.20 (0.90–1.60)	1.26 (0.96–1.66)
10	0.51 (0.29–0.91)	0.58 (0.35–0.96)	0.70 (0.44–1.13)	1.65 (1.17–2.33)	1.29 (0.96–1.74)	1.36 (1.02–1.81)
11	0.44 (0.24–0.80)	0.52 (0.30–0.88)	0.64 (0.39–1.05)	1.81 (1.27–2.60)	1.39 (1.02–1.89)	1.46 (1.08–1.97)
12	0.38 (0.20–0.70)	0.47 (0.27–0.81)	0.59 (0.35–0.98)	2.00 (1.37–2.91)	1.49 (1.08–2.06)	1.56 (1.14–2.13)
13	0.32 (0.17–0.61)	0.43 (0.24–0.75)	0.54 (0.32–0.92)	2.21 (1.49–3.27)	1.60 (1.15–2.23)	1.66 (1.20–2.29)
14	0.27 (0.14–0.53)	0.40 (0.22–0.71)	0.52 (0.3–0.89)	2.43 (1.62–3.65)	1.70 (1.20–2.39)	1.74 (1.24–2.43)
15	0.24 (0.12–0.47)	0.38 (0.21–0.69)	0.50 (0.29–0.88)	2.66 (1.74–4.04)	1.77 (1.25–2.53)	1.80 (1.27–2.54)
16	0.21 (0.10–0.43)	0.37 (0.20–0.68)	0.50 (0.28–0.89)	2.87 (1.86–4.43)	1.82 (1.27–2.62)	1.83 (1.28–2.61)
17	0.19 (0.09–0.40)	0.37 (0.19–0.69)	0.52 (0.29–0.93)	3.04 (1.94–4.77)	1.84 (1.26–2.67)	1.83 (1.27–2.63)
18	0.18 (0.09–0.39)	0.37 (0.19–0.72)	0.54 (0.30–0.99)	3.15 (1.98–5.03)	1.81 (1.23–2.66)	1.79 (1.23–2.61)
19	0.18 (0.08–0.39)	0.38 (0.20–0.75)	0.58 (0.31–1.07)	3.17 (1.96–5.13)	1.74 (1.17–2.60)	1.73 (1.17–2.55)
20	0.18 (0.08–0.41)	0.40 (0.20–0.79)	0.62 (0.33–1.17)	3.07 (1.86–5.05)	1.64 (1.09–2.48)	1.65 (1.10–2.46)
21	0.20 (0.09–0.45)	0.40 (0.20–0.82)	0.65 (0.34–1.26)	2.84 (1.69–4.76)	1.53 (0.99–2.35)	1.56 (1.03–2.36)

## Discussion

Yingjisha County in Kashgar region of Xinjiang is one of the regions with high incidence of TB in China. The results of descriptive statistical analysis showed that there were more female than male with TB cases in Yingjisha County, and a high prevalence among people ≥ 65 years of age, which was consistent with the results of a study by Jiang et al.^21^. It is suggested that the elderly population is key for TB prevention and control in Yingjisha County.

Temperature is an important meteorological factor affecting the incidence of TB, and Spearman correlation analysis in this study showed that among the meteorological factors, temperature had the most significant effect on the incidence of TB, showing a positive correlation with the incidence of TB, which was consistent with the results of the study in Hunan Province^22^. However, the results of the studies in various regions were not the same, such as North China, where the incidence of TB was negatively correlated with temperature^23^, and Jiangsu Province, where temperature had no significant effect on the incidence of TB^24^. This may be caused by environmental differences in meteorological factors due to geographical differences, or it may be caused by the problem of covariance caused by r_s_ > 0.8 between meteorological factors^25^. Therefore, in this paper, we used DLNM to control for the effects of confounding factors and explored the association between average daily temperature, average daily relative humidity, average daily wind speed and the incidence of TB.

The results of DLNM analysis presented that the effect of temperature on TB showed a non-linear relationship, and low temperature significantly increased the risk of developing TB, which was consistent with previous studies. Feng, F et al. indicated that the extremely low temperature has a significantly greater effect on respiratory diseases than extremely high temperature^26^. Chaw et al. investigated the association between climate variables and pulmonary TB incidence in Brunei-Muara district, Brunei Darussalam,and they observed positive but delayed relationship between PTB incidence and minimum temperature^27^. With higher RR values at a lag of 4 days for all groups, and in the discussion stratified by gender, the RR of developing TB reached its maximum at lag 21 days, − 15 °C for the male and female groups, which were respectively reported as 2.00, 95% CI (0.68–5.82) and 2.40, 95% CI (0.78–7.36), respectively; in the discussion stratified by age, the RR reached its maximum at lag 21 days, − 15 °C in the 36–64 and ≥ 65 age groups, reported as 2.58, 95% CI (0.84–7.96) and 1.99, 95% CI (0.65–6.09), respectively, and in the ≤ 35 age group at lag 4 days, -15℃ had the greatest RR, reported as 2.00, 95% CI (1.13–3.57). High temperatures were protective against TB transmission, this is in line with the findings noted by Chen et al. that extreme heat was associated with reduced PTB risk (RR = 0.982, 95% CI:0.966–0.998)^28^. The cumulative effect of hypothermia reached its maximum at lag 21 days, and the susceptibility of males was significantly higher than that of the female population, which may be attributed to the fact that males are the backbone of all sectors of society, have a lot of social engagements, and are more susceptible to TB because of the higher frequency of smoking and alcohol consumption and lack of physical exercise^29^. The susceptibility to hypothermia is significantly higher in people ≤ 35 years of age than in people over 35 years of age. On the one hand, this may be related to the social life environment of this age group, because the main force of this age group is adolescents, and adolescents gather in more confined environments for long periods of time, and low temperatures in winter will lead to a decrease in the frequency of window opening and ventilation, which will affect the probability of exposure to Mycobacterium TB; on the other hand, it may be due to the fact that temperatures directly change the indoor and outdoor activity time of susceptible and infected groups, which will affect the probability of exposure to probability of exposure to Mycobacterium TB^4^.

Unlike temperature, the risk of TB incidence in Yingjisha County had a hazardous effect at lag 7 days with increasing average daily relative humidity, a result consistent with Jiayuguan City^30^, and different from studies in Hunan and Wenzhou, Zhejiang^31,32^. Under high relative humidity conditions, the RR values of the groups were higher around lag 6 days, and in the discussion stratified by gender, the RR for the occurrence of TB in the male group reached a maximum at lag 6 days, 92%, reported as 1.08, with a 95% CI (0.95–1.23), and that of the female group reached a maximum at lag 0 days, 70%, reported as 1.05, 95% CI (0.88–1.25); in the discussion stratified by age, the RR in the ≤ 35 years group was maximal at lag 4 days, 92%, reported as 1.24, 95% CI (1.02–1.50), in the 36–64 years group was maximal at lag 5 days, 92%, reported as 1.08, 95% CI (0.93–1.26), and in the ≥ 65 years group it was maximal at lag 21 days, the maximum at 8%, reported as 1.15, 95% CI (0.83–1.59), and low relative humidity had a protective effect on the transmission of TB, with RR significantly greater than 1 at lag 21 days, 14 days, and 21 days for the male, ≤ 35 years, and ≥ 65 years groups, while cumulative RR was significantly less than 1 at lag 21 days, 14 days, and 21 days, reported as 0.61, respectively, 95% CI (0.39–0.97), 0.58, 95% CI (0.34–1.00) and 0.58, 95% CI (0.36–0.93), respectively. This may be due to the fact that relative humidity can affect health by influencing the body's circulatory system and increasing susceptibility to infectious diseases^24^, while relatively high humidity allows droplet nuclei containing Mycobacterium TB in a range of sizes in diameter to remain in the air for longer periods of time^33^, which may also increase the spread and prevalence of TB disease.

Under high wind speed conditions, the RR values of the groups were higher when the wind speed reached a maximum value of 5.2 m/s, around lag 16 days. In the discussion stratified by gender, the RR for the occurrence of TB in the male group was the greatest at lag 16 days, reported as 1.17, 95% CI (0.69–1.98), and that in the female group was the greatest at lag 15 days, reported as 1.43, 95% CI (0.86–2.35); in the discussion stratified by age, the RR was greatest at lag 4 days in the ≤ 35 years old, reported as 1.43, 95% CI (0.78–2.65), at lag 15 days in the 36–64 years old, reported as 1.27, 95% CI (0.78–2.07), and at lag 17 days in the ≥ 65 years old, with the RR reported as 1.47, 95% CI (0.81–2.67), and low wind speeds at lag 7 days and 14 days were protective against TB transmission. For wind speed, the higher the wind speed, the higher the risk of TB incidence. This is in line with the findings of Zhang et al. and Nie et al. Zhang et al. found that temperature and wind speed positively affected the incidence of TB by increasing the concentrations of fine particles can be inhaled and SO_2_^9^. Nie et al. stated that high wind speed could increase the risk of PTB^34^. On the one hand, it may be because Mycobacterium TB is mainly transmitted by air, and the higher wind speed is more conducive to bringing Mycobacterium TB from one place to another, accelerating the transmission of Mycobacterium TB. Yingjisha County is located in the desert region of Xinjiang Uygur Autonomous Region. Its unique geographical location will cause more dust and Mycobacterium TB in the air when the wind speed increases, and accelerate the incidence rate of TB. On the other hand, wind speed may cause temperature fluctuations, while higher wind speed may lead to lower temperature, the corresponding lower temperature will lead to increased relative humidity, and higher relative humidity can make Mycobacterium TB more comfortable to stay in the air, thus indirectly increasing the incidence of disease.

Due to the fact that the meteorological data obtained in this study are only average data, therefore, this study did not analyze the impact of the observed meteorological factors in the morning and evening on the incidence rate of TB. Only the impact of average data indicators was analyzed, which may result in bias in the research results. This is also the limitation of this study. In the future, we will further collect data to conduct a detailed analysis of the impact of morning and evening meteorological factors on TB and further analyze the effects of environmental factors such as atmospheric pressure, precipitation and air pollution indicators on pulmonary TB.

## Conclusions

Based on our study, each meteorological factor has more or less influence on the incidence of TB, and the risk of incidence decreases gradually with the increase of average daily temperature, has a hazardous effect within a certain humidity range with the increase of average daily relative humidity, and increases gradually with the increase of average daily wind speed. The average daily temperature, average daily relative humidity and average daily wind speed in Yingjisha County are one of the factors influencing the patients with latent TB, and meteorological factors play a role in the incidence and prevalence of TB in the population, not necessarily directly, but perhaps indirectly by influencing various aspects of the incidence of the disease (the living habits of the public, the ways of transmission, and the susceptible people's own physical fitness), and affecting the distribution of TB disease in the population. Therefore, based on the findings of this paper, people should take appropriate preventive measures. For example, under low AT conditions, especially at – 15 °C, women and middle-aged and elderly people should be reminded to keep warm and try to avoid staying in the cold environment for a long time. It is recommended to conduct health examination in advance, detect problems and take preventive measures. At a high RH environment (92%), when going out, avoid staying in humid environment, especially in confined space; in special weather with high average daily WS (5.2 m/s), ventilation management in public places should be strengthened to ensure air circulation and reduce the retention time of pathogens in the air. In addition, it is suggested that the whole population should do a good job in personal health management, maintain good living habits and diet structure, and enhance their own immunity. The results of this paper can provide a scientific reference for the TB prevention and control education of relevant CDC departments.

## Data Availability

The data that support the findings of this study are available on request from the corresponding author. The data are not publicly available due to privacy or ethical restrictions.
